# Molecularly Imprinted Polymer-Based Sensors for the Detection of Skeletal- and Cardiac-Muscle-Related Analytes

**DOI:** 10.3390/s23125625

**Published:** 2023-06-15

**Authors:** Serge Ostrovidov, Murugan Ramalingam, Hojae Bae, Gorka Orive, Toshinori Fujie, Takeshi Hori, Yuji Nashimoto, Xuetao Shi, Hirokazu Kaji

**Affiliations:** 1 Department of Diagnostic and Therapeutic Systems Engineering, Institute of Biomaterials and Bioengineering (IBB), Tokyo Medical and Dental University (TMDU), Tokyo 101-0062, Japan; ostrovidov.bmc@tmd.ac.jp (S.O.); hori.bmc@tmd.ac.jp (T.H.); nashimoto.bmc@tmd.ac.jp (Y.N.); 2 Institute of Tissue Regeneration Engineering, Dankook University, Cheonan 31116, Republic of Korea; rmurug2000@dankook.ac.kr; 3 Department of Nanobiomedical Science, BK21 NBM Global Research Center for Regenerative Medicine, Dankook University, Cheonan 31116, Republic of Korea; 4 Mechanobiology Dental Medicine Research Center, Dankook University, Cheonan 31116, Republic of Korea; 5 UCL Eastman-Korea Dental Medicine Innovation Center, Dankook University, Cheonan 31116, Republic of Korea; 6 School of Basic Medical Science, Institute for Advanced Study, Affiliated Hospital of Chengdu University, Chengdu University, Chengdu 610106, China; 7 Department of Metallurgical and Materials Engineering, Atilim University, 06830 Ankara, Turkey; 8 School of Basic Medical Sciences, Binzhou Medical University, Yantai 264003, China; 9 Institute of Precision Medicine, Medical and Life Sciences Faculty, Furtwangen University, 78054 Villingen-Schwennigen, Germany; 10 KU Convergence Science and Technology Institute, Department of Stem Cell and Regenerative Biotechnology, Konkuk University, Hwayang-dong, Kwangjin-gu, Seoul 05029, Republic of Korea; hojaebae@konkuk.ac.kr; 11 NanoBioCel Group, Laboratory of Pharmaceutics, School of Pharmacy, University of the Basque Country UPV/EHU, 01006 Vitoria-Gasteiz, Spain; gorka.orive@ehu.eus; 12 Bioaraba, NanoBioCel Research Group, 01009 Vitoria-Gasteiz, Spain; 13 Biomedical Research Networking Centre in Bioengineering, Biomaterials and Nanomedicine (CIBER-BBN), 01006 Vitoria-Gasteiz, Spain; 14 School of Life Science and Technology, Tokyo Institute of Technology, Yokohama 226-8501, Japan; t_fujie@bio.titech.ac.jp; 15 Living System Materialogy (LiSM) Research Group, International Research Frontiers Initiative (IRFI), Tokyo Institute of Technology, Yokohama 226-8501, Japan; 16 National Engineering Research Center for Tissue Restoration and Reconstruction, South China University of Technology, Guangzhou 510006, China; shxt@scut.edu.cn

**Keywords:** molecularly imprinted polymers (MIP), biomaterials, sensors, skeletal muscle, cardiac muscle

## Abstract

Molecularly imprinted polymers (MIPs) are synthetic polymers with specific binding sites that present high affinity and spatial and chemical complementarities to a targeted analyte. They mimic the molecular recognition seen naturally in the antibody/antigen complementarity. Because of their specificity, MIPs can be included in sensors as a recognition element coupled to a transducer part that converts the interaction of MIP/analyte into a quantifiable signal. Such sensors have important applications in the biomedical field in diagnosis and drug discovery, and are a necessary complement of tissue engineering for analyzing the functionalities of the engineered tissues. Therefore, in this review, we provide an overview of MIP sensors that have been used for the detection of skeletal- and cardiac-muscle-related analytes. We organized this review by targeted analytes in alphabetical order. Thus, after an introduction to the fabrication of MIPs, we highlight different types of MIP sensors with an emphasis on recent works and show their great diversity, their fabrication, their linear range for a given analyte, their limit of detection (LOD), specificity, and reproducibility. We conclude the review with future developments and perspectives.

## 1. Introduction

**Figure 1:**
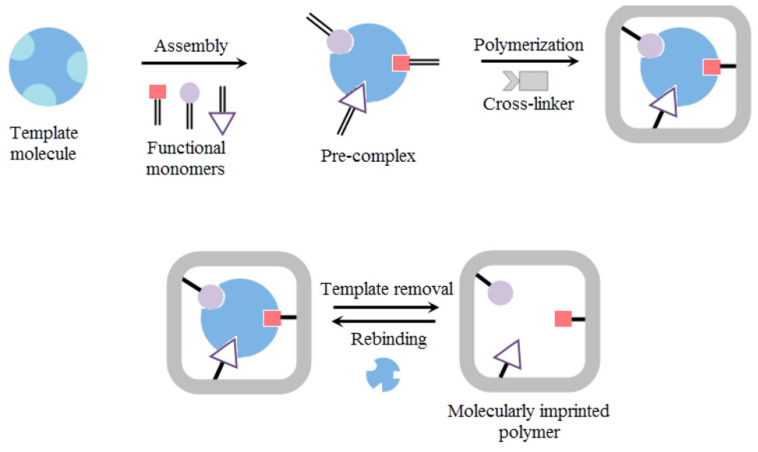
Schematic showing the preparation of a molecularly imprinted polymer (MIP) and the template detection. Reproduced from [20] with permission (open access).

Biomolecules, such as antibodies, DNA, enzymes, and aptamers, are very valuable in detection, sensing systems, and biological analysis [1,2,3]. Especially, antibodies are routinely used in biological assays and sensors because of their high affinity and selectivity. However, they are expensive and can easily lose their functionality, and their lifetime is limited [1,4]. Interestingly, molecular imprinting allows for the generation of artificial molecular recognition sites in a polymer. To this end, suitable monomers are polymerized in the presence of a template. After the removal of this template, the polymer matrix retains recognition cavities specific to this template with size, shape, and chemical functionalities complementary to it (Figure 1). These molecularly imprinted polymers (MIPs) are easy to make in large quantities, and they are cost-effective, robust, reusable, have a prolonged shelf-life, and can be fabricated for virtually any target molecules [1,5]. Furthermore, their properties can be tuned due to the high number of monomers available. Because of their high affinity and selectivity, MIPs have found many applications in different fields, such as affinity separation (possible resolution of racemic) [1,6] and purification [7], catalysis [8,9], enzyme inhibition [10], protein crystallization [11], immunoassays [12], chemosensing [13,14], drug delivery [15,16,17], drug development [7], and bioimaging [18,19].

Molecular imprinting was first introduced by Polyakov in the 1930s [21], and important advancements were proposed in the work of Wulff in the 1970s and later in the 1980s in the work of Mosbach. Two main approaches for molecular imprinting are possible. The first approach, introduced by Wulff in 1972, is the covalent approach in which the template binds to the polymer through reversible covalent bindings [22]. The advantage is that the binding sites are more homogeneous, whereas the disadvantages are that the template removal is more difficult and the rebinding of the template during detection takes longer. The second approach is the noncovalent approach, introduced by Mosbach, which is the most used because of its ease of use [7,23]. In this approach, the template binds to the polymer through noncovalent bindings (e.g., ionic interactions, hydrogen bonds, van der Waals interactions, hydrophobic interactions, and metal-ion chelating interactions). The advantages are that the template can be easily removed, allows for the imprinting of a wider range of compounds, and the rebinding of the template during detection is fast. The disadvantage is that the binding sites may be less homogeneous. A third approach, introduced by Whitcombe in 1995, has been described and is a hybrid approach between the covalent and the noncovalent methods [24]. In this approach, covalent bindings are used during the imprinting, while noncovalent bindings are used during the rebinding of the template. Thus, the imprinting is more precise and homogenous, whereas the template rebinding is fast. Several polymerization methods have been used to fabricate MIPs, among which are free radical polymerization (FRP), controlled radical polymerization (CRP), sol-gel process, electropolymerization, and photopolymerization [25]. Free radical polymerization is a common method used to fabricate MIPs and requires the use of a radical initiator with a template and monomer [26,27]. However, the control of the reactions of propagation and termination is difficult, resulting in different polymer chain lengths and branching. In controlled radical polymerization, a certain control of the reaction of propagation is obtained by fast activation/deactivation cycles, resulting in similar polymer chain lengths [28,29]. To this end, different approaches have been used, such as atom transfer radical polymerization (ATRP) [30,31,32], nitroxide-mediated polymerization (NMP) [33], iniferter-mediated polymerization [28,34], and reversible addition–fragmentation chain transfer (RAFT) [35]. In the sol-gel process, the monomer, template, crosslinker, and initiator are dissolved in a solvent (continuous phase), but upon initiation, the polymer formed is insoluble in the solvent and, thus, precipitates. Then, these tiny polymer particles act as seeds, and the polymerization progresses by the adsorption of monomer. This process allows for the formation of regular spherical polymeric nanobeads [36]. In emulsion polymerization, two non-miscible phases are used (oil in water or water in oil) to make an emulsion with the monomer, template, surfactant, and initiator. Upon polymerization, MIP nanobeads (10–100 nm) can be obtained [37]. With emulsion core-shell polymerization, a core (e.g., glass, latex, and magnetic nanobeads) is used as a seed upon which an MIP shell is polymerized. The advantage is the production of homogeneous nanoMIPs that are controlled in size and in the increase in surface-area-to-volume ratio [38]. Electropolymerization is also often used to fabricate MIPs on electrodes because of its ease of use [39]. The major advantage is that electropolymerization allows for the precise control of the thickness and morphology of the polymeric film that is formed, which has an effect on the accessibility to the binding sites and improves the reproducibility of the imprinting process. Furthermore, electropolymerization allows for the superior adhesion of the polymer on the transducer surface by adsorption. In photopolymerization, the monomer, template, and photoinitiator are subjected to UV or visible light, such as red, blue, and green (with camphroquinone, eosin Y, and methylene blue) [25]. The technique is easy and allows for the formation of very-thin polymeric films.

**Figure 2:**
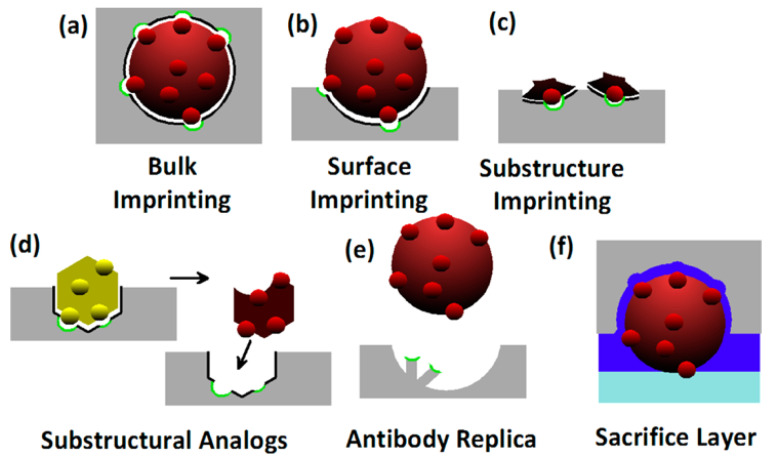
Examples of imprinting techniques used for the fabrication of MIP sensors. (**a**) Bulk imprinting with template fully embedded in the polymer, (**b**) surface imprinting with template partially embedded in the polymer, (**c**) epitope imprinting, which only uses a substructure of the targeted molecule as a template, (**d**) dummy imprinting, which uses an analog of the targeted molecule as a template, (**e**) imprinting of immunoglobulin, and (**f**) a variant of surface imprinting using disaccharide and polymeric thin film coating. Reproduced from [43] with permission. © 2014 by the American Chemical Society.

Among the materials often used to fabricate MIPs by polymerization [25,40] are functional monomers, such as acrylamide (AAm), methyl methacrylate (MMA), methacrylic acid (MAA), glycidylmethacrylate (GMA), aniline, pyrrole, and N-isopropylacrylamide (NIPAM); crosslinkers, such as ethylene glycol dimethacrylate (EGDMA) and ethylene dimethacrylate (EDMA); and free radical initiators, such as 2,2′-azobis(isobutyronitrile) (AIBN), and 4,4′-azobis(4-cyanovaleric acid) (ACVA). To induce polymerization, UV, visible light, and heat may be used. Furthermore, electropolymerization is also often used with MAA, polythiophene (PTh), polypyrrole (PPy), poly(3,4-ethylenedioxythiophene) (PEDOT), o-phenylenediamine (o-PD), polyvinylpyrrolidone (PVP), polyamine (PA), polyaniline (PANI), dopamine, and dimethylaminoethylmethacrylate (DMAEMA) [41]. Several imprinting processes are possible (Figure 2). In bulk imprinting, the template is fully incorporated into the polymer matrix. The imprinted polymer is usually ground and sieved to obtain access to the imprinted sites, to easily remove the template, and to obtain a similar particle size (usually micrometric size) with a high surface-to-volume ratio [42,43]. In surface imprinting, the template is partially incorporated into the polymer and, therefore, the binding sites form at the surface of the polymer [42]. In this category, we can also mention the surface stamping method, which uses a template stamp pressed onto a polymerizing film [44]. In epitope imprinting, a short peptide sequence is used as a template, and the binding sites at the surface of the polymer are selective to the template. Similarly, antibody epitope-imprinted polymers can be used for the binding of a whole protein that bears the epitope [45,46]. In nanoimprinting, nanosize structures (e.g., nanoparticles, quantum dots, graphene oxide, and nanotubes) are imprinted with the template. These nanoimprinted MIPs (or N-MIPs) have higher surface-area-to-volume ratios than bulk MIPs and have improved binding site accessibility, binding capacity, and binding kinetics [1,47]. In solid-phase imprinting, the template is first immobilized on a solid substrate (e.g., glass beads), and the MIP is polymerized on this fixed template. Then, the unreacted monomers and low-affinity MIPs are washed away at low temperature (~20 °C). The high affinity MIPs are released with washes at a high temperature (~60 °C) [48]. This process allows for the reuse of the fixed template and yields MIPs with high specificity but usually in smaller amounts. In surface grafting, the surface of the substrate is functionalized by chemical modification through covalent bonding [49]. These chemical groups then offer anchoring points for the deposit and growth of MIPs. To evaluate the imprinting efficiency of a newly fabricated MIP, a nonimprinted polymer (NIP) should be fabricated in the same conditions as those for the fabrication of the MIP but without the template [50]. This NIP is used as a control to assess the binding capacity and the selectivity of the interactions between the MIP and the template, since these interactions should be specific to the MIP and not to NIP. The imprinting factor (IF), which is the ratio between the binding capacities of the MIP on the NIP, could be used to characterize the MIP [50].

**Figure 3:**
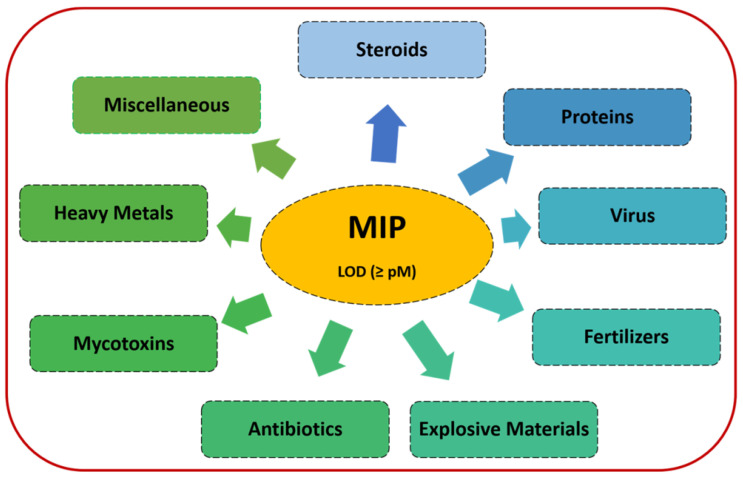
Examples of target analytes detected with the use of an MIP sensor. Reproduced from [27] with permission. © 2022 by the authors (open access).

Polymers can be imprinted with various types of templates (Figure 3). Thus, polymers have been imprinted with small molecules, such as gallic acid, caffeine, quercetin, single amino acid, formaldehyde, and dopamine [51,52], and with proteins, such as antibodies [53], human serum albumin [54], immunoglobulin G [55], cytochrome c [56], troponin [57], viral proteins [58], DNA [59], aptamer [60], whole virus [61,62,63], bacteria [64], spores [65], and living cells [66,67].

**Figure 4:**
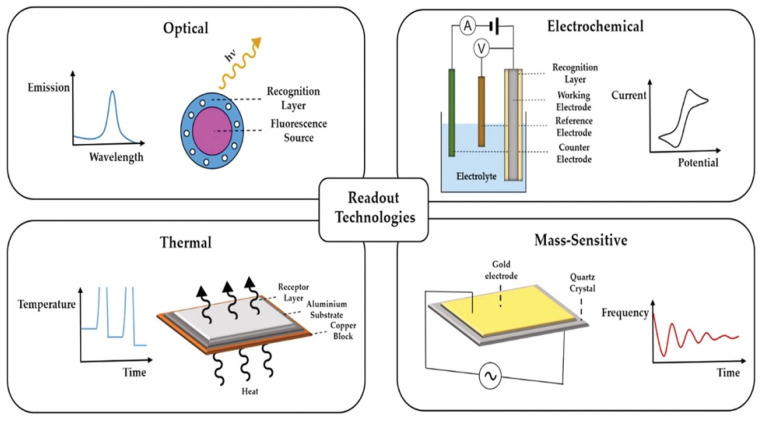
Examples of read-out technologies coupled with MIPs. Reproduced from [69] with permission. © 2023 by the authors (open access).

Sensors are composed by a recognition element that detects the targeted analyte coupled to a transducer, which converts the detection event into a quantifiable signal (Figure 4). For MIP electrochemical sensors, four types of transducers can be used: amperometric transducers, which evaluate changes in the current; potentiometric transducers, which evaluate changes in the potential; conductometric transducers, which evaluate changes in the conductometry; and impedimetric transducers, which evaluate changes in the impedance [68]. Furthermore, MIP-surface plasmon resonance (SPR) sensors evaluate the plasmon resonance frequency, which is related to the refractive index of a thin metallic layer under incident wavelength [69]. Other optical sensors can use fluorescent MIP quantum dots (QCs) and monitor the decrease in fluorescence when the template binds to the imprinted cavities [70]. Moreover, MIP sensors were combined with surface-enhanced Raman scattering (SERS) sensors [71]. In addition, the heat transfer method (HTM) is also often used with MIP sensors to evaluate the template detection [72]. Another type of MIP sensors used is quartz crystal microbalance (QCM) for which the resonant frequency changes when the analyte binds to the MIP, increasing the mass of the crystal [4]. QCM- and SPR-based sensors often have very-low LOD in the pM range [1]. Similarly, MIP sensors combined with surface acoustic wave technology use the decrease in wave velocity by the amount of mass as a controller [73]. Furthermore, MIP nanoparticles were immobilized on the cantilever of an atomic force microscopy (AFM) probe used as a mass sensor for the detection of ciprofloxacin [74].

**Figure 5:**
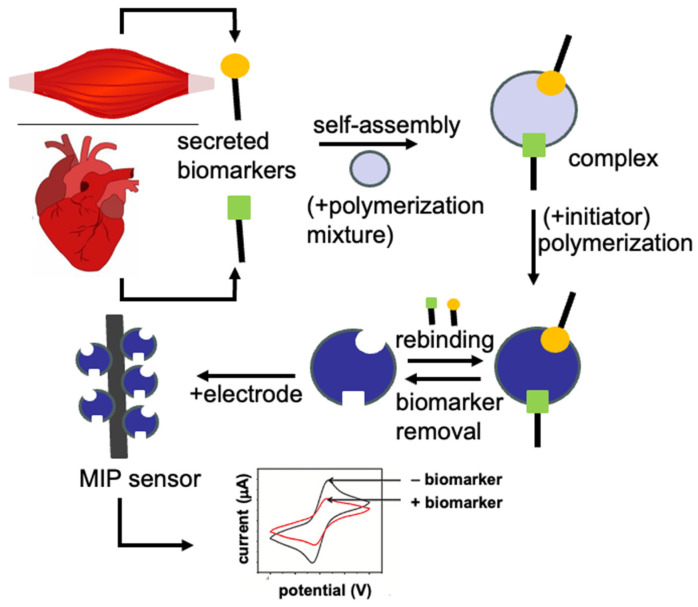
MIP formation for skeletal- and cardiac-muscle-related analytes. A secreted biomarker from skeletal muscle or cardiac tissues is used as a template and mixed with a mixture of polymerization (monomer, crosslinker, and initiator) to form the complex. Then, the initiator is activated by UV or heat, and the polymerization takes place. The template is removed with a desorption solution to form the MIP with free imprinted cavities, which can bind to the biomarker again. This MIP is coupled to a transducer (an electrode in this figure) and the binding of the template is monitored.

In the following section, we highlighted different MIP sensors fabricated for the detection of skeletal- and cardiac-muscle-related analytes. Therefore, the templates used for the fabrication of these MIP sensors are skeletal or cardiac muscle biomarkers [75]. These biomarkers are specific of a biological state (healthy or pathologic) and are usually used to establish a diagnosis or to monitor the results of a pharmacotherapy. The general fabrication method (Figure 5) for these MIP sensors consists in the polymerization of functional monomer(s) around one of these skeletal or cardiac muscle biomarkers used as a template to be imprinted in the presence of a crosslinker [76]. After the removal of the template with a desorption solution, specific cavities for this template remain in the imprinted polymer. Then, the imprinted polymer is used as a receiver and is coupled with a transducer to obtain a sensor. The development of new MIP sensors to this field is important for the clinics (improvement of diagnosis and clinical studies), for personalized medicine (self-monitoring), for drug discovery (when drugs are tested on skeletal or cardiac tissue models), and for skeletal and cardiac tissue engineering to evaluate the functionalities of the engineered tissues. Therefore, in this review, without being exhaustive of all MIP sensors that have been fabricated for the detection of skeletal- and cardiac-muscle-related analytes, we provide an overview of the different types of MIP sensors with an emphasis on recent publications and on their fabrication and their characteristics. We show their great diversity (e.g., impedimetric, potentiometric, colorimetric, and thermal analysis), the different imprinting methods that were used for their fabrication, their linear range for a given analyte, their limit of detection (LOD), and their specificity and reproducibility. Our aim in this review is to collect as many of the different targeted analytes related to skeletal and cardiac muscle tissues and organize them in alphabetical order.

## 2. Detection of Skeletal and Cardiac Muscle Analytes with MIPs

### 2.1. Analytes in Human Skeletal Muscles

**Figure 6:**
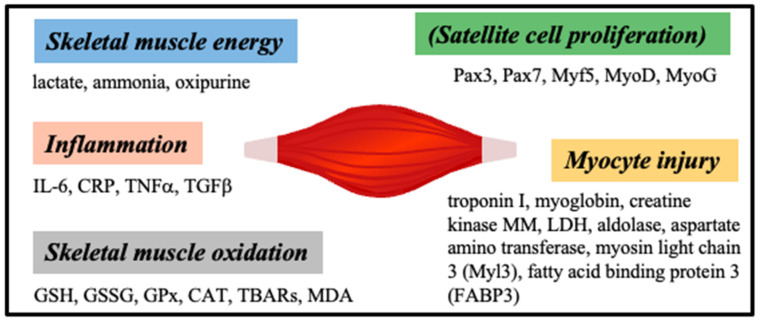
Schematic showing different skeletal muscle biomarkers.

Skeletal muscle injury or muscle disease can be caused by various factors, such as genetic, drug induced, trauma, disease progression, and aging. Among skeletal muscle biomarkers (Figure 6), creatine kinase (CK) and aspartate transaminase (AST) have been routinely used, but they lack sensitivity and tissue specificity (for example, they are shared with the cardiac muscle); therefore, the search for new skeletal biomarkers is a dynamic field [75,77].

- ***Baclofen***

Tarannum and Singh fabricated an MIP sensor for the detection of baclofen (4-amino-3-p-chlorophenylbutyric acid), which is a skeletal muscle relaxant, using water soluble monomers. They activated silica beads with (3-aminopropyl)triethoxy silane (APES) and grafted onto these amino-terminated silica beads N,N-methylenediacrylamide (MBA) followed by ethylene glycol 400 (PEG 400) and 1,3-propane sultone with baclofen (MIP) or without (NIP). After removing the template with four washes of water at 50 °C under 600 rpm, stirring for 10 min, the rebinding of baclofen in the imprinted cavities was monitored with spectrophotometry after elution, and the optimized time for target binding was 20 min. The sensor response was linear in the range 0.1–1 mg/mL of baclofen with an LOD of 0.125 μM. The sensor was selective to baclofen in the presence of interferants (e.g., chlorobenzene, aminobenzoic acid, phenylalanine, aniline hydrochloride, and phenyl acetic acid), and the IF was 1.70 [50,78].

- ***Bisphenol A***

Bisphenol A is an environmental pollutant, which has deep effects on skeletal muscles, impairing the metabolism of glucose, inducing oxidative stress, mitochondrial dysfunctions, and favoring type 2 diabetes [79,80,81]. Kong et al. combined a colorimetric sensor and MIP sensor to fabricate a microfluidic paper-based colorimetric MIP sensor for the detection of bisphenol A (BPA). The paper-based colorimetric sensor included two layers (sheets 1 and 2) of rectangular cellulose papers with patterns generated with wax through a stencil. These patterned paper layers were warmed in an oven at 130 °C for 150 s to melt the wax and to obtain hydrophobic barriers in the paper. ZnFe_2_O_4_ microparticles were used as a peroxidase mimetic to generate a colorimetric reaction by reacting with H_2_O_2_ and producing a hydroxyl radical (^•^OH), which oxidized 3,3′,5,5′-tetramethylbenzidine (TMD), producing the color change. The MIP membrane was formed on one microzone of sheet 1 by the bulk polymerization of a mixture of AAm (monomer), BPA (template), EGDMA (crosslinker), benzoin ethyl ether (BEE, initiator), ZnFe_2_O_4_, polyethylene glycol (PEG), styrene, and solvents placed under UV for 3 h. The sensor was washed with ethanol and then with methanol/acetic acid (9:1) to remove the template. TMD was applied on a microzone of sheet 2 that comes into contact with the below sheet 1 with the MIP membrane/ZnFe_2_O_4_ area. H_2_O_2_ was then added to generate the colorimetric reaction. Without BPA, H_2_O_2_ has easy access through the binding sites to ZnFe_2_O_4_, and the reaction generated radical hydroxyls, which oxidized TMD, and the color changed from grey to blue. With BPA, the binding sites are occupied, and H_2_O_2_ has less access to ZnFe_2_O_4_, and the TMD is less oxidized. Thus, the grey intensity decreases with the increase in BPA, as quantified by Photoshop software. The sensor was tested with BPA 100 nM–1 nM with the optimized parameters of pH 4 (acetate buffer of 0.2 M), 37 °C, H_2_O_2_ (0.5 M), binding time with BPA of 30 min, and time for colorimetric reaction of 10 min. The sensor response was linear in the range 10–1 μM of BPA with an LOD of 6.18 nM. The sensor showed selectivity for BPA when tested in the presence of interferants (e.g., tertbutyl hydroquinone (TBHQ), diethylstilbestrol (DES), hydroquinone (1,4-DHB), and phenolphtalein (PP)). The sensor also showed an acceptable reproducibility when tested with 500 nM BPA, since the relative standard deviation (RSD) for five MIP sensors from five different batches was 6.9%, and the RSD for five MIP sensors from the same batch was 8.4%. In addition, the sensor showed a good repeatability when tested to detect 60 nM of BPA four times after the removal of the target with methanol/acetic acid (9:1) [82]. Although the sensor was efficient, the binding analysis when MIP was immersed in 1–100 nM BPA showed that the binding sites in the MIP were not uniform, with one type of binding sites with high affinity and the other types with low affinity. Furthermore, Wang et al. wrote an interesting review on the fabrication and the advantages of the combination of colorimetric sensors and MIP sensors to obtain molecularly imprinted colorimetric sensors (MICSs) [83]. Furthermore, Hamed and Li wrote a review on MIP-based sensors for bisphenol A [84].

- ***Bromisoval***

Bromisoval (BVU) is a sedative and muscle relaxant commonly used in clinics. It also appeared in some cosmetics. However, elemental bromine can accumulate in the body and be toxic. Zhu et al. fabricated an MIP sensor to detect bromisoval. They first synthetized NiO/NiFe_2_O_4_ nanocubes with an anti-spinel type by the calcination of NiFe-PBA at 450 °C for 2 h. Then, the ZnCo-ZIF (zeolitic imidazolate frameworks (ZIFs)) formed around NiO/NiFe_2_O_4_ by in situ growth in methanol with zinc nitrate hexahydrate, 2-methylimidazole, and cobalt nitrate hexahydrate for 20 h at RT. The obtained NiO/NiFe_2_O_4_@ZnCo-ZIF was then mixed with thioacetamide under hydrothermal sulfidation to obtain the core-shell double heterogeneous nanocage NiO/NiFe_2_O_4_@Zn_0.76_Co_0.24_S NCs. A glassy carbon electrode (GCE) was then coated with an aqueous suspension of NiO/NiFe_2_O_4_@Zn_0.76_Co_0.24_S and dried to obtain NiO/NiFe_2_O_4_@Zn_0.76_Co_0.24_S/GCE. The MIP/NiO/NiFe_2_O_4_@Zn_0.76_Co_0.24_S/GCE sensor was obtained by the immersion of NiO/NiFe_2_O_4_@Zn_0.76_Co_0.24_S/GCE in a polymerization mixture of BVU (template), β-cyclodextrin (β-CD), and glutathione (GSH, monomers), phosphate buffer pH 7, and electropolymerization by cyclic voltammetry (CV). The optimized parameters were BVU, β-CD, GSH ratio of 1:1:1.3, and 12 scanning cycles at 65 mV/s. The template removal was obtained by the immersion of the MIP sensor in methanol/acetic acid (9:1). The binding of the BVU to the imprinted cavities was monitored with differential pulse voltammetry (DPV), and the optimized binding time was 10 min in Britton–Robinson buffer pH 9.2 (optimized buffer and pH). The sensor response was linear in the range of 0.006–3.7 μM of BVU with an LOD of 0.28 nM. The sensor showed specificity to BVU when tested in the presence of interferants (e.g., carbromal, bromhexine, ambroxol, ibrotamide, 4-bromoisoquinoline, tiotropium bromide, uric acid, glucose, bovine serum albumin, Cl^−^, NO_3_^−^, SO_4_^2−^, and Ca^2+^). Furthermore, an MIP2 sensor fabricated without GSH showed a peak current density lower than the MIP sensor fabricated with BVU + GSH, showing that GSH significantly increased the electrochemical activity of BVU and promoted the electron transfer. Moreover, the NIP-sensor (fabricated without BVU) showed a weak peak current density. The sensor also showed good reproducibility, repeatability, and stability, since the RSD for six different MIP sensors was 2.83%, the RSD for five measurements with one MIP sensor was 3.57%, and the remaining peak current density of the MIP sensor after 2 weeks of storage at 4 °C was 96.15%. Finally, the RSDs were less than 5% when the sensor was used to detect BVU in serum, facial mask, sunscreen, and moisturizing lotion [85].

- ***Creatinine***

Creatinine is a byproduct of the muscle metabolism formed by the decomposition of creatine phosphate, and it is excreted in the urine [86]. Normal levels of creatinine in urine are in the range of 0.5–1.5 mg/mL and in the blood in the range of 0.7–1.2 mg/mL [87]. Hassanzadeh et al. fabricated an MIP sensor for creatinine detection by capping near-infrared-emitting Ag2S-functionalized COOH quantum dots (QDs) with TMSPMA, and then with the MIP layer formed by the radical polymerization of MAA (monomer) grafted on QDs and EGDMA (crosslinker) in the presence of creatinine (template) and AIBN (initiator). The template was removed with ethanol washes. The binding of creatinine in the imprinted cavities was monitored by fluorescence quenching using UV-vis fluorescence spectrophotometry, and the optimal time and pH for target binding was 15 min and pH 8. The response of the sensor was linear in the range of 0.15 mg/mL–0.5 mg/mL, and the LOD was 0.6 mg/mL. The sensor was selective to creatinine in the presence of interferants (creatine, L-tyrosine, and N-hydroxysuccinimide (NHS)), and the IF was very high with a value of 1233 [70]. In another study, Nontawong et al. fabricated an amperometric MIP sensor for the simultaneous detection of creatinine and 8-hydroxy-2′-deoxyguanosine (8-OHdG), which is a molecular marker of oxidative stress and DNA damage [88]. A solution of creatinine (template) in dimethyl sulfoxide (DMSO) was mixed with MAA (monomer) and N,N-(1,2 dihydroxyethylene)bisacrylamide (DHEBA, crosslinker) and AIBN (initiator). Then, copper oxide (CuO) nanoparticles were added to the mixture to form a CuO-MIP sensor. The same process was used but without a template to form CuO-NIP. Template removal from CuO-MIP was obtained by sonication in water for 30 min. In the next step, platinum nanoparticles decorated with reduced graphene oxide (PtNPs-rGO) were synthetized, and then imprinted with dopamine (monomer), guanosine (template), and ammonium persulfate (APS, initiator) to obtain PtNPs-rGO-MIP. Template removal from PtNPs-rGO-MIP was obtained after washes with methanol/acetic acid (9:1) and water. Then, a dual sensor was fabricated by packing a homogeneous paste of graphite-PDMS powder, CuO-MIP nanoparticles, PtNPs-rGO-MIP, and mineral oil into a glass tube to form a working electrode. The binding of creatinine and (8-OHdG) to the imprinted cavities was monitored with amperometry; there were no optimal times for target binding, because the binding for both analytes was very fast, whereas the optimal pH for target binding was pH 7.5. The response of the sensor was linear in the ranges of 0.5–150 μM for creatinine and 5 nM–50 μM for 8-OHdG, and the LODs were 77 nM and 0.8 nM for creatinine and 8-OHdG, respectively. The sensor showed a good selectivity to creatinine and 8-OHdG in the presence of interferants (e.g., ascorbic acid, guanosine, serotonin, dopamine, urea, and NHS) and good response stability over 14 days. When tested with real serum and urine samples, the results were in good agreement with values from a clinical laboratory with relative differences of <5% [89]. Moreover, Alizadeh and Mousavi fabricated an MIP sensor for the detection of creatinine. However, since creatinine is a hydrophilic molecule, it cannot be dissolved in nonpolar solvents. The researchers found that the complexation of copper ions (Cu^2+^) with creatinine makes it soluble in acetonitrile, allowing to overcome this limitation. Therefore, the MIP was prepared by mixing CuBr_2_ with creatinine in acetonitrile. Then, MAA (monomer), EGDMA (crosslinker), and AIBN (initiator) were added to the mixture and kept at 60 °C for 12 h. A carbon paste was formed by mixing graphite, MIP nanoparticles, and melted N-eicosane. This carbon paste (2 × 3 mm cylindrical) was integrated into an electrode, and CV was used to monitor the copper ion oxidation signal as an indirect evaluation of creatinine in phosphate buffer at pH 5 with fixed Cu II (10 μM). Interestingly, it was observed that the copper ion signal was amplified in the presence of creatinine with the MIP electrode and not with the NIP electrode. The sensor response was linear in the ranges of 0.1–1 μM and 1–10 μM with an LOD of 59 nM. This linearity result showed that there are two types of binding sites in the MIP with different affinities for the analyte. The sensor was tested with similar compounds of creatinine (e.g., caffeine, uric acid, and melamine) and showed selectivity to creatinine. A good agreement was obtained with the results from human plasma samples spiked with creatinine evaluated with the sensor and with HPLC [90]. Furthermore, Sergeyeva et al. fabricated a colorimetric MIP sensor for the detection of creatinine by the immersion of a poly(vinylidene difluoride) (PVDF) membrane (pores of 0.22 μm) into a mixture of creatinine (template), itaconic acid (IA, monomer), MAA (monomer), 2-acrylamido-2-methyl-1-propanesulfonic acid (AMPSA, monomer), and MBA (crosslinker) subjected to UV (365 nm) for 10 min. The template was removed by hot methanol in Soxhlet for 2 h. The binding of creatinine to the imprinted cavities was revealed by reaction with picric acid, which forms an orange-red-colored complex (Jaffe test). A linear range was established for creatinine concentrations in the range of 0.25–2.5 mM, with an LOD of 250 μM. The sensor showed high selectivity to creatinine with negligible interactions with interferants (e.g., creatine, glucose, sarcosine, and urea). Furthermore, the sensor was stable over 1 year of storage at RT. When the MIP sensor was tested in comparison with HPLC for the detection of creatinine, the results obtained were in excellent agreement [91]. This very long stability of the MIP sensor is impressive and a significant advantage of the MIP (plastic antibody) over the use of natural antibodies. Finally, Panasyuk-Delaney et al. fabricated an MIP sensor for the detection of creatinine using photopolymerization under UV of 2-acrylamido-2-methyl-1 propane sulfonic acid (AMPS, monomer), benzophenone (initiator), and MBA (crosslinker) in the presence of creatinine (template, MIP) or not (NIP) on a hexadecanethiol-coated gold electrode. After the removal of the template in distilled water at 50 °C for 1 h, the MIP sensor was evaluated for capacitive chemical sensitivity to creatinine with electrical impedance spectroscopy (EIS). Creatinine binding was monitored with the decrease in the electrode capacitance. Linearity was observed from 0 to 600 μM with an LOD of 10 μM. The sensor was highly selective to creatinine, with no response when tested with other compounds (e.g., sodium chloride, creatine, urea, and glucose) and remained efficient over 6 months when stored in air at room temperature (RT) [92].

- ***Glucose***

Omidvar et al. fabricated an MIP sensor for the detection of glucose. First, an MIP was fabricated by mixing acrylic acid (monomer), glucose (template), EGDMA (crosslinker), and 4,4′-azobis (4-cyanopentanoic acid) (ACPA, initiator). Polymerization was initiated by exposure to UV. The resulting MIP was milled into a fine powder, which was mixed with poly(methyl methacrylate) (PMMA), and 20 μL of this solution was applied to the tip of a stub resonator. The template was removed by washing with water for 5 h. The MIP-coated tip was fixed in the sensing area integrated in a microfluidic device to control the flow of liquid, and the changes in the resonant frequency due to the glucose fixation to the binding sites was monitored. The different solutions of glucose with concentrations ranging from 0.5 mg/mL–to 4 mg/mL were tested at a flow rate of 5 mL/min. The results showed a shift in the peak response with an increase toward higher frequencies with the increase in glucose concentration. The sensor was selective to glucose, since no shift in frequency was observed when NIP, and solutions of mannose and galactose were used. The sensor response was linear in the range of 0.5 mg/mL–to 4 mg/mL of glucose with a lower limit of detection (LLD) of 24 pg/mL [93]. In another study, Sehit et al. fabricated an MIP sensor for the detection of glucose by electropolymerization of o-PD (monomer), glucose (template), and gold nanoparticles (AuNPs) on a bare gold electrode. The template removal from the polymer was obtained by incubation in NaOH 0.3 M for 10 h. The binding of glucose in the imprinted cavities was monitored with amperometry, and the time for target binding used was 30 min. The sensor response was linear in the range of 1.25–320 nM of glucose with an LOD of 1.25 nM. The AuNPs MIP sensor was selective to glucose, since no signal was observed in the presence of interferants (e.g., sucrose, dopamine, starch, and bovine serum albumin), except for sucrose. Furthermore, when 40 nM glucose was incubated with AuNPs NIP, no crossreactivity was observed. The sensor’s response was also stable over a 40-day storage period. When tested with real serum samples, the sensor detected glucose with an LOD of 1.25 nM [94]. Interestingly, Crapnell et al. combined electrospinning and an MIP sensor for the detection of glucose. Imprinted PPy particles were obtained by mixing D-glucose (template), pluronic 123 (micelle generator), and pyrrole (monomer), and then ferric chloride hexahydrate. Then, the MIP was washed with methanol/water (50:50) to remove the pluronic. The template was removed by refluxing with methanol/water (50:50). NIP was fabricated in the same way but without D-glucose. Then, MIP was ground into a fine powder, mixed at 12.1% with acid formic, and nylon 6,6 monomer, and then electrospun to obtain a web of nylon with the imprinted microparticles. This electrospun web was placed in a device with an increasing concentration of D-glucose (0.05–1 mM) in PBS, and the thermal resistance, R_th_, was monitored. The results showed that the thermal resistance increased from 3.5 ± 1.3 to 34.6 ± 1.4% with the increase in D-glucose (0.05–1 mM). The sensor was selective to D-glucose, because no change in the thermal resistance was observed when NIP was used with different concentrations of D-glucose and when MIP was used with interferants, such as fructose, galactose, lactate, and urea. When tested on sweat samples, the sensor showed a linear response in the range of 0.1–0.8 nM of D-glucose and an LOD of 0.12 nM [95]. This study with the combination of electrospun nanofibers and MIP opens the possibility for the fabrication of wearable fabrics with integrated sensors. Moreover, Caldara et al. developed an MIP sensor for the detection of glucose by the bulk imprinting of a polymerization mixture of AAm (monomer), EGDMA (crosslinker), 2,2′-azobis(2-methylpropionitrile) (initiator), and D-glucuronic acid (dummy template) in DMSO at 65 °C for 10 h. The optimized composition was 8:12:40:48.5 in DMSO, respectively. The bulk MIP was ground, washed with methanol, dried, and then milled and sieved to obtained microparticles smaller than 100 μm in size. The template was removed by Soxhlet extraction with a mixture of acetic acid/methanol (1:6) for 16 h, followed by another extraction in pure methanol for 16 h. A planar sensor electrode was fabricated by immobilizing MIP particles by micro-contact printing on an aluminum plate modified with a polyvinylchloride (PVC) adhesive layer, and the modified aluminum plate was placed on a copper block (heater) in a flow cell. The binding of glucose to the imprinted cavities was monitored with the heat transfer method (HTM). The sensor showed a linear response to glucose in PBS in the range of 0.0194–0.33 mM with an LOD of 19.4 μM. The sensor was tested in the presence of interferants (e.g., fructose, sucrose, and lactose) and showed specificity to glucose. It was mentioned that the other analytes could interfere with the sensor at low concentrations (≤0.05 mM). Furthermore, when the MIP and NIP responses to glucose were compared, the IF was 2.95. The sensor was used to detect glucose spiked in human urine samples, and its response was linear in the range of 44.4–330 μM, with an LOD of 44.4 μM [72]. This study presented a dummy imprinting technique, which uses a template with a similar structure, shape, size, and function, instead of the real template, because glucose does not form strong ionic interactions with monomers, as it has basic or acidic functionality. Furthermore, Caldara et al. wrote an excellent review of the use of MIP for glucose monitoring [96].

- ***Glutathione***

Glutathione (glycine-cysteine-glutamic acid, GSH) is a major antioxidant protecting cells against reactive oxygen species (ROS), but it is also involved in cell signaling and in detoxification. Antioxidative defenses match the rates of free radical production, which is correlated to oxygen consumption by tissues. Skeletal muscles with high oxidative capacities also have a high GSH content, and physical exercise that induces oxidative stress also induces an increase in antioxidant enzyme activities [97,98]. Konishi et al. fabricated a MIP potentiometric sensor to detect GSH. They modified a graphite electrode with the deposition of a thin film obtained by plasma polymerization and immersion in a mixture of sodium dodecyl sulfate (SDS, surfactant), 2,2′-azobis(4-methoxy-2,4-dimethylvaleronitrile) (V-65, radical initiator), dibutyl phtalate (plasticizer), and water for 24 h at RT. Then, the MIP was formed by the immersion of the modified electrode in a polymerization mixture of polyvinyl alcohol (PVA), GSH (template), MAA (monomer), EDMA (crosslinker), V65 (radical initiator), toluene, and heat treatment (70–75 °C for 12 h). The GSH/MAA ratio was optimized to 2:32. The template removal was obtained by the immersion of the MIP/thin film/graphite electrode in water. The potential responses in the presence of GSH 1–500 μM were monitored with a potentiometer and the sensor response after 25 min incubation with different GSH concentrations showed linearity in the range of 10–200 μM. The sensor showed a good selectivity to GSH in the presence of cysteine and glycine, but a poor selectivity in the presence of glutamic acid. Furthermore, the IF was 8.21 [99].

- ***Growth hormone***

Bohlooli et al. fabricated an MIP sensor for the detection of the human growth hormone (HGH). MIP was formed on a magnetic Fe_3_O_4_ nanoparticles/glassy carbon electrode (GCE) with the electropolymerization of aniline (monomer, 0.1 M) and HGH (template, 1.25 mg/mL). The template was removed by incubation in H_2_SO_4_ 1 M for 1 h. The binding of HGH to the imprinted cavities was monitored with square wave voltammetry (SWV) and showed after 30 min incubation a linear response in the range of 0.1–100 ng/mL. The sensor showed good reproducibility and stability with 91% remaining current after 45-days of storage at 20 °C. The sensor also showed a high selectivity to HGH when incubated with interferants (e.g., BSA, casein, glucose oxidase, creatinine, urease, progesterone, cortisol, and testosterone). Tested with real human serum samples spiked with HGH 1 ng/mL at pH 6.42, the sensor response was linear in the range of 0.1–100 ng/mL, with an LOD of 60 pg/mL [100].

- ***Insulin***

Insulin is a protein of fifty-one amino acids and a mass of 6 kDa. It is an anabolic hormone that regulates glucose homeostasis. Insulin stimulates glucose uptake by skeletal muscles [101]. The normal level of insulin in blood is 12–150 pM [102]. Zidaric et al. fabricated a MIP sensor for the detection of insulin. The MIP was formed with the electropolymerization of a mixture of pyrrole (monomer) and insulin (template) at a ratio of 1:4 in PBS (0.01 M) at pH 7.2 on the surface of a screen-printed carbon electrode (SPCE) with CV at 50 mV/s and 10 cycles. The template was removed using electrocleaning with CV at 50 mV/s and 25 cycles. The binding of insulin (5 mL) to the imprinted cavities was monitored with SWV in the presence of [Fe(CN)6]^3−/4−^ (5 mM) and showed, after 15 min of incubation, a linear response in the range of 20–70 pM with an LOD of 1.9 pM. The selectivity of the MIP and NIP sensors to insulin was assessed and, while the peak current decreased with the increase in insulin concentration with the MIP sensor, the NIP sensor did not show any linear response. Then, three different MIP sensors were tested with real samples (from commercial pharmaceutical test samples 0.6 mM) after dilution and the RSD was 7.23%. The reproducibility of the sensor was tested on six replicate measurements with different sensors and with 20 pM insulin and 5 mM of [Fe(CN)6]^3−/4−^ and the RSD obtained was 5.92%. The repeatability of the sensor was also tested with one MIP sensor and ten measurements, and the RSD obtained was 5.13% [103]. Furthermore, Wardani et al. fabricated an MIP sensor for the detection of insulin. A gold electrode was modified with carboxylated multiwalled carbon nanotubes (f-MWCNTs, 2 mg/mL) drop-casted on its surface and allowed to dry for 3 h at RT to form f-MWCNTs/AuE. A polymerization mixture was prepared with chitosan (monomer), acrylamide (AM, monomer), and MBA (crosslinker) in PBS at pH 5.3 and sonicated for 30 min. Then, 10 mL of this polymerization mixture was mixed with 5 mL insulin (template), 1 mL APS, and 0.1 mL N,N,N′,N′-tetramethylethylenediamine (TEMED), and 4 mL of this mixture was drop-casted on f-MWCNTs/AuE and frozen at −10 °C for 5 h to form the MIP cryogel/f-MWCNTs/AuE. The template was removed with the immersion of the MIP sensor in acetic acid/ethanol (9:1) for 2 h. The MIP sensor was integrated in a flow system and a flow rate of 100 mL/min was chosen. The binding of insulin in the imprinted cavities was monitored with SWV. The sensor’s response was linear, in the range 0.050–1.40 pM with an LOD of 33 fM. The MIP sensor was tested in the presence of interferants (e.g., glucose, uric acid, creatinine, dopamine, ascorbic acid, human serum albumin, carcinoembryonic antigen, and alphafetoprotein), and showed a high selectivity to insulin with nearly no response for interferants. The reproducibility of the sensor was evaluated with six different MIP sensors on five different concentrations of insulin ranging from 0.050 to 0.40 pM. The RSDs obtained were in the range 2.90–7.83%, showing that the fabrication method was highly reproducible. The stability of the MIP sensor was investigated and the results showed that the sensor was stable for up to forty-six detections of insulin with an RSD of 1.4%. Beyond that, the sensor’s response decreased below 90% because of the loss of the cryogel. Moreover, the stability of the modified electrode was assessed after storage in a dry condition at RT, and the electrode was stable up to 10 weeks. Insulin was also evaluated in ten diluted real serum samples with the MIP sensor compared to the Elecsys insulin assay used in a hospital, and the results obtained by both methods are in great agreement [102]. In another study, Kartal et al. developed an MIP-based QCM sensor for the detection of insulin. First, the gold-chip surface of a QCM sensor was modified with allyl mercaptan. Then, insulin (template) and N-methacryloyl-(L)-histidine methylester (MAH) were mixed at RT for 2 h. Another mixture of AIBN (initiator), EGDMA (crosslinker), and 2-hydroxyethyl methacrylate (HEMA, monomer) was prepared, in which the insulin/MAH solution was added to obtain the prepolymer solution. A total of 5 mL of prepolymer solution was dropped on the thiol modified gold surface of the QCM and spin-coated. The polymerization was performed under UV for 45 min at RT. The poly(hydroxyethyl methacrylate)-N-methacryloyl-(L)-histidine-methyl ester (PHEMAH) sensor obtained was washed with ethanol and dried. The template was removed with an aqueous solution of NaCl (0.5 M). Different aqueous solutions of insulin (0.008–10 ng/mL) were applied to the MIP QCM sensor and the increase in insulin concentration induced an increase in the sensor’s response with a plateau reached in 20 min. The MIP QCM sensor’s response was linear in the insulin range of 1.38 pM–1.72 nM with an LOD of 0.27 pM. The sensor also showed good selectivity toward insulin when tested with interferants (e.g., glucagon and aprotinin), while the NIP QCM sensor showed much lower responses than the MIP QCM sensor. The sensor was also used to detect insulin in spiked diluted (1/5) artificial plasma samples and the results were compared to those obtained with the ELISA method. The comparison showed that the MIP QCM sensor had a higher accuracy than the ELISA method. Furthermore, the insulin adsorption/desorption cycles were repeated four times and the results show that the sensor displays reproducible mass shifts with no decrease in the binding capacity. In addition, the sensor’s response was stable after 1 week of storage, and showed a 30% decrease after three months of storage [104].

- ***Interleukin-1b***

IL-1b is an inflammatory cytokine secreted from immune cells, such as monocytes and macrophages. IL-1b increases in the skeletal muscle after physical exercise [105]. Choi et al. fabricated an MIP sensor for the detection of IL-1b. The working electrode surface of an SPCE was modified with the deposition of a thin layer of poly(o-phenylenediamine) using the electropolymerization of o-PD in acetate buffer at pH 5.2. Then, the MIP was formed with the electropolymerization of a mixture of o-PD (monomer), chromotrope 2R (C2R, monomer), and IL-1b (template) in PBS at pH 7.4 using CV at 150 mV/s and two cycles. The template was removed with the immersion of the MIP sensor in an aqueous solution of NaOH (0.1 M) for 90 min. The binding of IL-1b at different concentrations (6 fM–60 nM) in PBS at pH 7.4 to the imprinted cavities was monitored with EIS, and with a binding time of 2 h. The sensor’s response was linear in the range of 0.1 pg/mL–1.05 ng/mL of IL-1b with an LOD of 0.23 pg/mL. The sensor showed a high selectivity to IL-1b in the presence of interferants (e.g., IL-6, TNFa, and IL-1a). The MIP sensor was also tested with real serum samples that had concentrations of IL-1b ranging from 6 fM (0.1 pg/mL) to 0.6 pM (10 pg/mL), and the R_CT_ values increased from 5231 Ω to 7141 Ω as the IL-1b increased [106]. This MIP sensor had a very low LOD (below the picogram); however, the template binding time (2 h) was too long.

- ***Interleukin-6 (IL-6)***

IL-6 is a major myokine secreted by skeletal muscles after exercise and consists of 184 amino acids, with a molecular weight of 26 KDa. It also acts on myoblasts and satellite cell proliferation, depending on its concentration [107,108]. In a recent study, Oliveira et al. created an interesting combination of microneedle arrays and MIP for the transdermal detection of IL-6. They fabricated polycarbonate microneedle arrays with pyramidal microneedles with a 1000 μm height, 600 μm square base, 20 μm tip, and 1200 μm gap between the needles. Each array was divided into four regions and metallized: two regions served as working electrodes and were coated with chrome and platinum, one region served as reference electrodes and was coated with chrome and silver, and one region served as counter electrodes and was coated with chrome and platinum. The MIP sensor was obtained with the electropolymerization of a mixture composed of 3-amino-phenylboronic acid (APBA, monomer), IL-6 (template), and PBS buffer pH 7.4. The template was removed by incubation in a solution of proteinase K (500 μg/mL) in PBS overnight at 40 °C. The binding of IL-6 (1 pg/mL–10 ng/mL) in artificial interstitial fluid (ISF) in the imprinted cavities was monitored with EIS, and the optimized time for target binding was 20 min. The sensor response was linear with the increase in the logarithm of IL-6 concentration over 1 pg/mL and the LLD was 1 pg/mL [109]. This combination of microneedles and MIP sensor is very interesting for transdermal diagnostic and monitoring because microneedles can be painless. In addition, they are also useful for drug delivery. In another study, Yaman et al. fabricated an impedimetric MIP sensor for the detection of IL-6. They synthesized peptide nanotubes (PNTs) by the self-polymerization of diphenylalanine (FF) and 1,1,1,3,3,3-hexafluoro-2-propanol (HFIP) at 60 °C for 1 h. After cooling at RT, they modified the surface of a screen-printed electrode (SPE) by the deposition of the PNT solution to form PNT/SPE. The PNTs exhibit micrometric length tubular structures on the SPE by scanning electron microscopy (SEM), which increase the surface of the SPE and allows for more binding sites to be created. The MIP was fabricated by mixing dopamine hydrochloride (Da, monomer), IL-6 (template), and Tris-HCl pH 8, overnight at RT under stirring. Then, this oxidized pDa solution was deposited on the modified electrode to obtain MIP(pDa)/PNT/SPE. The template was removed by washing with a NaCl (1 M) solution for 30 min at RT, which is a soft method for the removal of a protein and increases the specificity of the sensor. The binding of IL-6 in the imprinted cavities was monitored with EIS and the increase in resistance. The sensor response was linear in the range of 1–200 pg/mL with an LOD of 0.25 pg/mL. The sensor had an IF of 8.7 and showed a high selectivity to IL-6 in the presence of interferants (e.g., IL-8, glucose, and BSA). The sensor was tested with spiked urine samples and the recovery values ranged between 99.04% and 104.9%, with a low RSD (<4.32%) [110]. Polydopamine has a strong adhesive property, which is interesting to link the MIP and the electrode. In addition, this study showed a soft method to remove the template and a high selectivity. Furthermore, Goncalves et al. electropolymerized an MIP solution on a screen-printed carbon electrode (SPCE). Pyrrole (monomer), pyrrole-2-carboxylic acid (Py-COOH, monomer), and IL-6 (template) were mixed at RT for 30 min (to establish hydrogen bonds between IL-6 and Py-COOH), which was then drop-casted onto the C-SPE and incubated for 5 min before electropolymerization with CV 50 mV/s and 10 cycles. Template removal was obtained by the immersion of the MIP/SPCE in oxalic acid dihydrate (0.05 M) for 3 h. The rebinding of IL-6 to the imprinted cavities was monitored with EIS and an incubation time of 30 min. The sensor response was linear in the range of 0.02 pg/mL–20 ng/mL with an LOD of 0.1 pg/mL. The sensor was tested with spiked serum samples and the sensor response was linear above 0.02 pg/mL [111].

- ***Lactate***

Lactate is mainly produced by muscle cells from glycogen and glucose, and its concentration increases during exercise to support the demand in energy. The lactate level in blood ranges from 0.5 to 1.5 mM at rest and can rise to 25 mM during physical effort [112]. Zhou et al. fabricated a wearable MIP amperometric sensor for lactate by modifying a SPCE with Ag-Au nanoparticles and the electropolymerization of a solution of 3-aminophenyl boronic acid (monomer), lactic acid (template), and NaCl. After the removal of the template with acetonitrile for 2 min, the detection of lactate in PBS at pH 7.4 was monitored by amperometry. The sensor response showed linearity in the lactate range of 1–220 μM and an LOD of 3 nM. Furthermore, the sensor was highly selective when tested against other compounds (e.g., urea, glucose, pyruvic acid, uric acid, KCl, NaCl, CaCl_2_, MgCl_2_, and NH_4_Cl) and resistant toward hard bending or twisting, showing reproducible response. The evaluation of lactate from the sweat of sportsmen after 30 min of exercise resulted in a mean value of 17.2 mM [113]. The homogeneous modification of SPCE using the Ag-Au nanoparticles (100–200 nm) enhances the electron transfer, improving the analytical signal. Moreover, Ben Moussa et al. fabricated a MIP sensor for the detection of lactate. Reduced graphene oxide (rGO) was prepared by dispersion of graphite oxide in water and sonication. Then, AgNO_3_ was added followed by the addition of 50 mL of *Allium sativum* (garlic) extract and the solution was stirred for 12 h at 80 °C to form reduced graphene oxide decorated with silver nanoparticles (rGO-AgNPs). The rGO-AgNPs was washed and dried at 50 °C. A colloidal solution of rGO-AgNPs in ethanol was prepared and 3 mL was drop-casted on a gold electrode and let to dry at RT. The MIP was formed on the modified electrode with the electropolymerization of aniline (monomer) and lactic acid (template) in HCl (0.5 M) and H_2_SO_4_ (0.2 M) with CV at a scan rate of 50 mV/s and 5 cycles. The template was removed with the immersion of the MIP/rGO-AgNPs/AuE in a mixture of ethanol/water (1:1) for 20 min. The binding of lactate in the imprinted cavities was monitored with CV at a scan rate of 50 mV/s in PBS (0.1 M) at pH 7.4 with 5 mM of [Fe(CN)_6_]^3−/4−^ used as a redox marker. The sensor’s response was linear in the range of 10–250 mM of lactate with an LOD of 0.726 mM. The MIP sensor was selective to lactate when tested in the presence of interferants (e.g., citric acid, ascorbic acid, vitamin E, and thiamine), with negligible current variation with interferants without lactate, and a 1.2% difference in the sensor’s responses with lactate only and the mixture of lactate with all interferants. Furthermore, seven MIP sensors were prepared and tested with 10 mM of lactate, and the MIP sensor showed reproducibility with no significant difference in the current variation, and with an RSD of 3.25%. In addition, the MIP sensor was stable with a current variation of only 3.9% after thirty cycles of adsorption/desorption of lactate. The MIP sensor was also tested with lactate (10 mM) solutions at different pH ranging from pH 5.2 to pH 10.5 and showed acceptable recoveries ranging from 93.9 to 102% with an RSD of 2.92%. Moreover, the MIP sensor was tested with diluted real serum samples, and the concentrations of lactate detected were within the normal range [114]. Furthermore, Mustafa et al. fabricated an MIP sensor for the detection of lactate. The MIP was fabricated using the bulk polymerization of a mixture of MAA (monomer), lactate (template), EGDMA (crosslinker), and AIBN (initiator) subjected to UV (365 nm) for 30 h at 4 °C. The obtained polymer was ground, milled, and sieved to obtain a powder with grains of 7 mm. The template was removed by successive washes with PBS. The detection of lactate was monitored with a UV-vis spectrophotometer for 60 min of incubation and then elution of the template in PBS. The sensor showed a linear response toward lactate in the range of 0.1–1.7 mM with an LOD of 0.162 mM and an IF of 6.86. The sensor was selective to lactate when tested with molecules with structural resemblance (malic acid, and sodium 2-hydroxybutyrate) with a three-fold higher retention of lactate than the interferants (63.5%, 22.6%, and 25.2%, respectively) [115]. Although the sensor is efficient, the incubation time with the template (1 h) is long. In another study, Pereira et al. fabricated an MIP sensor for the detection of lactate. They deposited an rGO film on a GCE by chronoamperometry. Then, gold nanoparticles (Au-NPs) were electrodeposited on the modified electrode with +0.5 V for 100 s. Finally, the MIP was obtained with the electropolymerization of o-PD (monomer) and lactate (template) in phosphate buffer at pH 5. The template was removed with immersion in acetonitrile for 60 s at RT. The binding of lactate (0.1–15 nM) to the imprinted cavities in phosphate buffer at pH 6 was monitored with differential pulse voltammetry (DPV) in the presence of potassium ferricyanide (K_3_[Fe(CN)_6_]), and the binding time used was 10 min. The peak current decreased with the increase in lactate concentration, and the MIP/AuNPs/rGO/GCE sensor response was linear in the ranges 0.1–1 nM and 1–15 nM with an LOD of 0.09 nM. Thus, the MIP sensor contains two types of binding sites with different affinities for the template. The sensor was selective to lactate with a higher response than with the interferants (e.g., malic acid, tartaric acid, butyric acid, acetic acid, glucose, and glycerol). The sensor also showed a good stability, since after 21 days of storage at RT, the electrode still displayed 89.7% of the initial current with an RSD of 3.2% [116]. Moreover, Mugo et al. fabricated a multilayered sensor of conductive nanoporous carbon nanotube-cellulose nanocrystal (CNC/CNT) film and poly(N-isopropylacrylamide) (pNIPAM) microgel. Lactate-imprinted poly(aniline)/polyphenylboronic acid (PANI/PBA) were coated onto pNIPAM to induce the pNIPAM microgel to respond to lactate instead of NaCl. Following the polymerization of the MIP, the template was removed with cyclic voltammetry electrochemical cleaning in phosphate buffer (0.1 M, pH 7). The lactate-imprinted PANI/PBA/pNIPAM/(CNC/CNT) sensor response showed linearity in the range of 1–25 mM of lactate with an LOD of 0.1 mM and a binding time of 5 min. The sensor was selective to lactate with no response to the interferants (e.g., urea, ascorbic acid, and glucose) as to NIP. Lactate was detected in real sweat samples with values of 6 mM. Moreover, the sensor was subjected to mechanical deformation (rolling) for the evaluation of its resilience and stability, and no degradation of the response to lactate was observed [117]. Responsive polymers are interesting for their switch between two states depending on their environment. In sweat, the main electrolyte is NaCl, which normally induces the collapse of pNIPAM, but the study showed that the coating of pNIPAM with MIP induced a new behavior by pNIPAM. In another study, Mugo et al. fabricated an array of PDMS microneedles coated with a conductive PDMS/carbon nanotubes (CNT)/cellulose nanocrystal (CNC) composite (PDMS/CNT/CNC) for multiplex sensing (pH, epinephrine, dopamine, and lactate). The modified microneedles were then silylated with 3-(trimethoxysilyl)propyl methacrylate (TMP) by drop casting, and then a layer of PANI/CNT/CNC/AgNP was electrodeposited to obtain PANI/CNT/CNC/AgNP@PDMS/CNT/CNC. The area of microneedles dedicated to lactate sensing was additionally coated with lactate-imprinted PANI-co-3-aminophenylboronic acid (PANI-co-3-APBA) gold nanoparticles (AuNPs) to form the MIP sensor (PANI-co-3-APBA/AuNP@PANI/CNT/CNC/AgNP@PDMS/CNT/CNC). Template removal was obtained by CV (45 cycles) in PBS. The response of the MIP sensor was linear in the range of 25–150 mM with an LOD of 0.07 mM. The sensor also showed selectivity to lactate in the presence of interferants (e.g., cortisol, uric acid, and ascorbic acid). The lactate level evaluated in human sweat samples after exercise was 139 mM, which is within the usual range [118]. This study is another example of a wearable sensor patch with microneedles for transdermal analyte analysis that allows for multiplexing by defining different areas of sensing in the microneedle array. In addition, Alizadeh et al. fabricated a potentiometric MIP sensor for the detection of lactate. They polymerized a mixture of allyl amine (monomer), lactic acid (template), EGDMA (crosslinker), and AIBN (initiator) in acetonitrile at 60 °C for 24 h. The template was removed by hydrolysis with a methanolic/KOH solution at 80 °C for 8 h. Then, a graphite rod electrode was coated by immersion for a few seconds in a mixture of 2.5% MIP nanoparticles in tetrahydrofuran (THF), 65% dibutylphtalate (DBP), 2% multiwalled carbon nanotubes (MWCNTs), 2% tetraphenyl phosphonium bromide (TPPB), and 28.5% polyvinyl chloride (PVC) powder to obtain an optimized functional-membrane-coated electrode. The lactate binding in the imprinted cavities was monitored by potentiometry at RT with a binding time shorter than 60 s. The sensor response was linear in the range of 1 μM–0.1 M with an LOD of 0.73 μM, and the potential response was constant at a pH of 5.0–8.0. It was observed that the conductivity of the electrode was best improved by MWCNTs rather than graphite or graphene particles. The evaluation of the potentiometric selectivity coefficients of the electrode for different anionic species (e.g., ascorbate, citrate, oxalate, cysteine, maleate, glutarate, sulfate, nitrate, and chlorate) showed that the sensor was selective to lactate ions; however, cysteine at high concentrations may interfere with it. No significant difference in the results obtained was observed when the sensor was compared to a titration method for the evaluation of lactate in milk and yoghurt samples [119]. Finally, Zaryanov et al. reported on the detection of lactate in sweat samples with an MIP sensor based on boronate-functionalized polyaniline developed on SPE by electropolymerization of 3-APBA (monomer) in the presence of lactate (template). The sensor response was linear in the range of 3–100 mM of lactate with an LOD of 1.5 mM and a response time of 2–3 min. Furthermore, the sensor’s sensitivity remained after 6 months of storage in a dry state at RT [120].

- ***Myoglobin***

Myoglobin is a hemic protein found in skeletal muscles and cardiac cells involved in oxygen transport and storage. Piloto et al. fabricated an MIP sensor for the detection of myoglobin using cadmium telluride quantum dots (QDs) capped with mercaptopropionic acid (MPA) and coated their surfaces with an MIP produced by the radical polymerization of acrylamide (monomer) and bisacrylamide (crosslinker) in the presence of myoglobin (template, MIP) or not (NIP, used as a control). After the removal of the template, the binding of myoglobin in the imprinted cavities was monitored using fluorescence quenching. The saturation of the imprinted cavities following the thermodynamic equilibrium with a stable fluorescence signal was reached within 30 min, when the sensor was incubated with 1.5 nM myoglobin. Tested with standard solutions of myoglobin with increasing concentrations, the QDs with an imprinted surface outperformed the QDs prepared with bulk imprinting. The sensor detected myoglobin concentrations from 571 to 0.304 pg/mL (95 pM to 50.6 fM) with an LOD of 0.045 pg/mL (7.6 fM) [121]. Furthermore, Karami et al. reported on an MIP sensor for the simultaneous detection of prostate-specific antigen (PSA) and myoglobin in human serum and urine samples. First, 3,3′-dithiodipropionic acid di(N-hydroxysuccinimide ester) (DSP) was self-assembled on an SPE. Then, myoglobin and PSA (templates) were deposited on the surface of the DSP-SPE to react for 24 h. Then, the imprinting process was performed with AAm (monomer) and MBA (crosslinker) in the presence of the templates on the DSP-SPE with ammonium persulfate. After the removal of the templates by incubation with oxalic acid (1 M) for 12 h, the binding of the targeted compounds on the sensor was monitored with EIS, and the optimal time and temperature for the target binding were 60 min and 25 °C, respectively. The MIP/DSP/SPE sensor response was linear in the range of 1 ng/mL–20 mg/mL of myoglobin with an LOD of 830 pg/mL. The sensor was selective to myoglobin in the presence of interferants (e.g., BSA, cortisol, epidermal growth factor receptor (EGFR), vascular endothelial growth factor (VEGF), human thrombin (HTR), human albumin serum (HAS), neuron specific enolase (NSE), carcinoembryonic antigen (AgCE), and thrombin antigen (AgTA)). Furthermore, an immunosensor specific to PSA was implemented on the MIP sensor with the use of magnetic microbeads decorated with MWCNTs and monoclonal anti-PSA antibodies, allowing for dual detection and quantification. The sensor was tested with spiked samples of serum and urine, and the results obtained were in good agreement with the results obtained with a reference method (ELISA). Furthermore, the sensor had a good stability over 1 month with a CV at less than 4.8% [122]. Moreover, Sullivan et al. studied the selectivity of MIPs fabricated from different acrylamide-based monomers (AAm, N-(hydroxymethyl)acrylamide (NHMAm), N-(hydroxyethyl)acrylamide (NHEAm), N,N-dimethylacrylamide (DMAm), and N-(Tris(hydroxymethyl)methyl)-acrylamide (TrisNHMAm)) to myoglobin. They compared experimental results to predictions via computer simulation. They obtained the following MIP classifications through the selectivity factor: NHMAm > TrisNHMAm > Aam > NHEAm > DMAm with 1.55, 1.48, 1.46, 1.38, and 1.32, respectively [123]. Furthermore, they fabricated thin-sheet MIP hydrogels by the compression of the polymerization mixture made of one of the previous acrylamide-based monomers, with myoglobin, (MBA) (crosslinker), and ammonium persulfate between two sheets of parafilm. The template was removed by soaking in SDS: acetic acid for 2 h. They observed a similar functionality to the bulk MIPs without the need for grinding and sieving [124]. In another study, Osman et al. fabricated an MIP-SPR sensor by synthetizing a myoglobin-imprinted poly(hydroxyethyl methacrylate-N-methacryloyl-L-tryptophan methyl ester) (poly(HEMA-MATrp)) nanofilm on the gold surface of a surface plasmon resonance (SPR) sensor. They used a microcontact imprinting technique. After the removal of the template by washing with ethylene glycol (1 M), the binding of myoglobin in the imprinted cavities was monitored in PBS with a pH of 7.4, and the dR% value reached a plateau within 25 min. The MIP sensor’s response was linear in a myoglobin range of 0.1–1 μg/mL and an LOD of 26.3 ng/mL. The MIP sensor was tested in the presence of interferants (lysozyme, cytochrome c, and BSA) and showed a high selectivity for myoglobin. In addition, the MIP sensor was tested with diluted serum samples, and the myoglobin concentration detected was 42.6 ng/mL, which was in good agreement with the result obtained with ELISA (58.3 ng/mL) [125].

- ***Myostatin***

Anti-myostatin monoclonal antibodies have been developed and used within a framework for muscular wasting disorders to inhibit myostatin in order to increase skeletal muscle mass. However, such myostatin-neutralizing antibodies (e.g., domogrozumab, landogrozumab, and stamulumab) are prohibited in sports. Torrini et al. fabricated an MIP sensor for the detection of anti-myostatin monoclonal antibodies. At first, they fabricated the MIP on a planar gold surface of an SPR chip by the bulk imprinting of norepinephrine (monomer) with the whole monoclonal antibody mAb-1 (GnRH1, mouse anti-human GNRH clone LHRH13–327.8) used as a template and incubated 5 h at 25 °C. The template was removed with 5% acetic acid and rinsed with water. The binding of IgG antibodies in buffer to the imprinted cavities was monitored with SPR for a binding time of 120 s and a flow rate of 5 μL/min. After each binding test, the MIP sensor was regenerated with a short pulse (24 s) of SDS 0.1% and 20 mmol/L HCl. However, when the selectivity of the MIP sensor was tested against different subclasses of IgG (IgG2, IgG3, and IgG4), the sensor showed a good selectivity to IgG1, but a crossreactivity with IgG4. Therefore, the research team moved toward an epitope-imprinting strategy to enhance the selectivity. To this end, norepinephrine (monomer) was polymerized on a planar gold surface in the presence of a short peptidic sequence (^439^KSLSLSPGK^447^, acronym FcC_H_3) directed against the Fc portion of the heavy chain of immunoglobulin (Ig) isotype G. The template was removed with 5% acetic acid and rinsed with water, as previously described. The sensor response was linear in the range of 0.63–10 μg/mL of mAb-1 at 25 °C with an LOD of 70 ng/mL. This new MIP sensor showed a higher selectivity to IgG1 with an IF of 13.5 and a reduced interactivity with IgG2, IgG3, and IgG4. Since FcC_H_3 is conserved in IgG subclasses (1, 2, 3, and 4), this difference in reactivity was attributed to conformational differences, allowing for the recognition of IgG1 only by the imprinted cavities. The sensor was tested with human serum samples. The anti-myostatin antibodies were extracted from the serum samples by magnetic beads conjugated to myostatin and directly injected into the SPR chip. The sensor response was linear in the range of 0–20 mg/mL of anti-myostatin antibodies with an LOD of 211 ng/mL [126].

- ***Ractopamine***

Ractopamine (RAC) is a β_2_-adrenergic agonist that promotes skeletal muscle growth. RAC may be abused as a livestock feed additive, and its accumulation in animal tissues may be of risk for humans. Roushani et al. fabricated a hybrid aptamer (Apt)-MIP electrochemical sensor for the detection of RAC. First, they prepared an Apt/RAC complex in TBST buffer by mixing at 37 °C for 1 h; then, they modified a glassy carbon electrode (GCE) by the electrodeposition of silver nanoparticles (AgNPs), and they added the Apt/RAC complex on AgNPs/CGE and allowed it to dry for 5 h. They saturated any free Apt sites with an RAC solution, and then they carefully removed unbounded Apt from the electrode with phosphate buffer at pH 7.4. In the second step, they electropolymerized an aqueous solution of dopamine onto the surface of the Apt/RAC/AgNPs/CGE. After, they removed the template (RAC) with a polar solution of ethanol/acetic acid/SDS (5 mg) stirred overnight. As a control, they also prepared the counterpart NIP, which was Apt/AgNPs/GCE. The binding of RAC in the imprinted cavities was monitored with EIS and the optimized time for the target binding was 60 min. The sensor response was linear for an RAC range of 1 fM–1902 nM, and the LOD was 330 aM. The sensor showed a high selectivity toward RAC, and the sensor’s response was reproducible (five different devices were tested), repeatable (five measurements with the same device), and stable (50 repetitive cycles, cyclic voltammetry). At least, the RAC concentrations were determined precisely in urine and blood samples loaded with 10^−1^, 10^2^, and 10^5^ pM RAC [127]. Although the template rebinding time is long (1 h), the sensitivity of the MIP sensor is impressive. Concerning this type of aptamer-MIP sensor, we inform readers of Ali and Omer (2022)’s review [60].

- ***Testosterone and derivatives***

Testosterone is a sex hormone and an anabolic androgenic steroid that can enhances sport performance [128]. Cimen fabricated an MIP SPR sensor to detect testosterone. Poly(HEMA-MAA) nanoparticles were obtained using the polymerization method of two-phase mini-emulsion. Testosterone (template) and MAA (monomer) were mixed for 2 h. Then, this mixture was added to the oil phase consisting of EGDMA and HEMA and stirred for 1 h. A first aqueous phase of polyvinyl alcohol (PVA), sodium dodecyl sulfate (SDS), sodium bicarbonate, and a second aqueous phase of PVA and SDS were prepared separately. The oil phase was added slowly to the first aqueous phase, which was homogenized by centrifugation at 6000 rpm for 30 min. Then, the second aqueous phase was added to the mixture and, finally, ammonium persulfate (initiator) and sodium bisulfite were added. The polymerization reaction was conducted at 40 °C and 500 rpm for 24 h. The gold surface of an SPR was functionalized with thiol groups with the incubation overnight of the SPR with allyl mercaptan at RT. Imprinted nanoparticles (62.96 nm) were dropped and homogeneously spread over the surface with spin coating, and then subjected to UV 20 min and 40 °C heating in an oven overnight. The template removal was obtained by washing with NaCl (0.1 M) for 3 min. The sensor’s response was linear in the range of 0.5–20 ng/mL of testosterone in an aqueous solution with an LOD of 0.049 ng/mL. The IF was 4.16, and the sensor showed selectivity to testosterone when used with interferants (e.g., b-estradiol and progesterone). Moreover, the MIP SPR sensor was tested on artificial urine samples spiked with testosterone (1 ng/mL and 2 ng/mL) for 6 min of application, and the LOD obtained was 0.038 ng/mL. The response of the sensor was also compared to the results obtained from ELISA, and both methods were consistent. Furthermore, the repeatability of the sensor was tested on four replicates (adsorption/desorption cycles) with 12 ng/mL of testosterone, and the recovery was 97.47% [129]. In another study, Liu et al. fabricated an MIP electrochemical sensor for the detection of testosterone. The MIP was fabricated on indium tin oxide (ITO) electrodes with the polymerization of a mixture of aniline (monomer), m-aminobenzenesulfonic acid (MSAN, monomer and dopant), testosterone (100 μg/mL, template), and ammonium persulfate (initiator). The template was removed with an aqueous solution of ethanol 5%. CV was used to evaluate the binding of testosterone at 0.01–5000 pg/mL in the imprinted cavities. The response of the MIP sensor to testosterone was linear in the range of 0.1–100 pg/mL, and the LOD was a few pg/mL. The poly(aniline-co-metanilic acid) film showed selectivity to testosterone with an observed current of 1200 μA/cm^2^, while with the interferants (17β-estradiol, progesterone, urea, and creatinine) and NIP polymer, the current was low (<20 μA/cm^2^). When the sensor was used with real urine samples, the concentrations of the detected testosterone were in the range of 0.33–9.13 ng/mL, with an average accuracy of 90% compared to a commercial analyzer [68,130]. Furthermore, Lee et al. used a four-channel potentiostat and MIP sensors for the simultaneous detection of four hormones: testosterone, cortisol, 17β-estradiol, and progesterone. The MIP polymers were fabricated on an SPE with the electropolymerization of a mixture of aniline (monomer), MSAN (monomer and dopant), and one of the templates (10 μg/mL) cortisol, testosterone, progesterone, or 17β-estradiol. The templates were removed by washes with an aqueous solution of ethanol 5%. Cyclic voltammetry was used to monitor the response of the sensor with different hormones at concentrations in the range of 0.001–1000 fg/mL. The responses of the sensors were linear for cortisol at 1–1000 ag/mL, testosterone at 1–1000 ag/mL, progesterone at 0.001–10 fg/mL, and 17β-estradiol at 0.001–1000 fg/mL, with LODs of 2 ag/mL, 10 ag/mL, 2.5 ag/mL, and 9 ag/mL, respectively. The sensors were used for the detection of these hormones in urine samples, and the values obtained were: cortisol, 19.3–60.5 ng/mL; testosterone, 0.14–3.37 ng/mL; progesterone, 0.7–2.7 ng/mL; and 17β-estradiol 28–288 pg/mL [131]. The sensors presented in this study are ultra-sensitive, with an LOD in the order of ag/mL.

Anabolic androgenic steroids are synthetic or natural variants of testosterone. They include nandrolone, oxandrolone, oxymetholone, stanozolone, trenbolone acetate, boldenone, and methandrostenolone, and they are agonists of the androgen receptor. They stimulate the growth of skeletal muscles and are not allowed in sportive competition [132]. Zhao et al. fabricated an MIP sensor for the detection of nandrolone (19-nortestosterone, (19-NT)). First, they modified a GCE with gold nanoparticles by electrodeposition. Then, the MIP was electropolymerized on the Au/GCE with CV at 40 mV/s and 40 cycles. The mixture for polymerization was tetrabutylammonium perchlorate, 2-aminothiophenol, and 19-NT (template). The template was removed by the incubation of the MIP/Au/GCE in methanol/acetic acid (ratio 9:1) for 120 min. Amperometry was used to monitor the functionality of the MIP sensor, and its response in the presence of nandrolone was linear in the range of 5–95 μM with an LOD of 3 nM. The MIP sensor was selective to nandrolone, and no interference effects were observed with interferants that tested 10-fold more concentrated (e.g., caffeine, 4-nitro-benzoic acid, cysteine, hypoxanthine, glucose, xanthine, dopamine, oxalic acid, ascorbic acid, uric acid, KCL, NaNO_3_, MgSO_4_, and CaCl_2_). Moreover, urine samples were tested for nandrolone, and perfect matches were observed between the results obtained with the sensor and those obtained with ELISA [133]. In another study, Bao et al. fabricated an MIP sensor for the detection of metandienone (MD), also known as methandrostenolone. They modified a GCE with a coating of graphene oxide (GO) in dimethylformamide (DMF). Then, the MIP was electropolymerized on the GO/GCE with a mixture of pyrrole (monomer), MD (template), and H_2_SO_4_ (0.1 M). The template was removed with incubation in oxalic acid dehydrate (0.05 M) for 30 min. Amperometry was used to monitor the functionality of the MIP/GO/GCE sensor, and the current increased with the increase in concentrations of MD. The sensor’s response was linear in the range of 0–2900 ng/mL of MD with an LOD of 70 pM. The sensor was selective to MD, and no interference effects were observed with interferants that tested 10-fold more concentrated (e.g., testosterone, glutamic acid, 19-NT, boldenone, stanozolone, keto-testosterone, methylboldenone, progesterone, methyltestosterone, b-estradiol, a-testosterone, acetaminophen, uric acid, dehydroepiandrosterone, a-nortestosterone, a-boldenone, keto-nortestosterone, and clenbuterol). The sensor was used to detect MD in urine samples, and the results obtained were approximately 5 ng/mL with RSD values between 3.15 and 4.73% [134]. Finally, Zheng and Lu fabricated an MIP sensor for the detection of stanozolone, which is a synthetic anabolic steroid. The surface of a GCE was modified by immersion in DMF with CNTs for 15 min and then dried under an infrared heat lamp. The MIP formed on the modified GCE by electropolymerization of a mixture of aniline (monomer) and stanozolol (template) in phosphate buffer (scanning rate 20 mV/s, 5 cycles). The template was removed by immersion in methanol/acetic acid (9:1) for 5 min. The amperometric current at 0.52 V in 0.1 M PBS at pH 7 increased with the increase in stanozolone, and the MIP/CNTs/GCE sensor showed a linear response in the range of 0–120 μM with an LOD of 9 pg/mL. The MIP sensor was tested in the presence of numerous interferants (e.g., ascorbic acid, dopamine, glucose, lactose, folic acid, cholesterol, uric acid, lactic acid, acetaminophen, dehydroepiandrosterone, codeine, norepinephrine, albumin, Fe^2+^, Ca^2+^, Zn^2+^, and Mg^2+^), and no significant electrocatalytic signal was observed in contrast to the clear amperometric increase with stanozolone. Stanozolone was evaluated in plasma samples from healthy bodybuilders, and the recovery ranged from 99.3% to 99.6%, whereas the RSDs ranged from 3.81% to 4.42% [135].

- *(Analytes in animal skeletal muscles)*

Antibiotics are often found in the food chain because of their direct use in livestock or their discharge into the environment. These antibiotics are direct potential threats to human health and may contribute to the emergence of pathogens with antibiotic resistance. Several MIP sensors for the detection of antibiotics have been fabricated, and in this section, we cited several examples. Enrofloxacin (ENRO) is an antibiotic used in livestock and in aquaculture. However, excessive ENRO residues in food may harm human health. In Europe, a regulation of 100 μg/kg for bovine, ovine, caprine, and poultry muscles has been established. Neng et al. combined a SERS sensor and MIP sensor to detect ENRO. A silver film was formed on the surface of copper rods (5 cm × 0.1 cm) with the immersion of copper rods in an aqueous silver trifluoroacetate solution (10 mg/mL) at 30 °C for 5 min (chemical reduction). The MIP sensor was obtained with the immersion of the AgNPs-copper rods in a polymerization mixture of MMA (monomer), dimethyl acrylate 1, 4-butylene glycol ester (crosslinker), phenyl silane (initiator), and ENRO (template) at 75 °C for 1 h. The template was removed by the immersion of the MIP/AgNPs/copper rods in 10% methanol acetate for 3 h. The sensor showed an intense SERS enhancement effect due to the narrow gaps formed among the adjacent silver nanoparticles (NP average size: 81 nm). The Raman peak intensity at 1629 cm^−1^ increased with an ENRO concentration increase from 0.001 to 0.1 μg/mL. The response of the sensor was linear in the range of log 10^−3^–log10^−1^ of ENRO, and the LOD was 25 pg/mL. The sensor showed selectivity to ENRO when it was tested with a mixture of ENRO and interferants (e.g., ciproflaxin, moxifloxacin, and norfloxacin), with no response in the Raman spectra without ENRO, as with the NIP sensor. The sensor was also used to detect ENRO in food. Minced pork was spiked with different concentrations (0.1, 0.05, and 0.02 μg/mL) of ENRO, the supernatants extracted were placed on the sensor for 120 min, and three measurements for each concentration were performed. The recovery rates ranged between 90.61 and 92.72% with an RSD of 2.42–5.16%, and these results were consistent with those obtained by HPLC evaluation [136]. The combination of SERS and MIP techniques is interesting because SERS has a high sensitivity, whereas MIP also has a high selectivity. In another study, the antibiotic targeted was florfenicol (FF). FF is regularly used prophylactically in animal farming (pig, bovine, and poultry) and especially in salmon farming. Caro et al. reported on a nanoMIP coated on a microwell plate for the detection of FF in salmon muscle. They fabricated the MIP using glass beads with epoxy groups and immobilized FF, which were incubated in a polymerization mixture of MAA (monomer), EGDMA and trimethylolpropane trimethacrylate (crosslinkers), benzyldithiocarbamate (initiator), pentaerythrioltetrakis (3-mercaptopropionate) (chain transfer agent), and acetonitrile. The mixture was subjected to UV for 2 min. After washings, the MIP-coated beads were placed in acetonitrile with poly(ethylene glycol) acrylate 1100 and irradiated for 30 s with UVA lamps to obtain an outer shell to ease the beads’ immobilization in a microwell plate. The template was removed by washes with acetonitrile at 22 °C. MIP or NIP in 0.2% polyvinyl alcohol (PVA) was dropped into each well of a 96-well plate and dried. After washing and blocking, a mixture of horseradish peroxidase (HRP), luminol enhancer, and stable peroxide was added to each well, and the absorbance was read at 450 nm with a microplate reader. A similar process was conducted with FF-conjugated HRP. Since the affinity of the binding sites in MIP is higher for free FF than for FF-conjugated HRP, the free FF concentration in a sample can be determined by the binding competition with FF-conjugated HRP, and the decrease in the fluorescence intensity was measured at 450 nm. The nanoMIP detected well the FF concentrations that ranged within 10–60 ng/mL (with an upper limit of 300 ng/mL), whereas concentrations ranging within and below 1.2–5 ng/mL were not discriminated. The nanoMIP was also tested with interferants (e.g., oxytetracycline (OTC), flumequine (FMQ), thiamphenicol (TPH), and florfenicol amine hydrochloride (FFA)), and the results showed a very low binding with them, and even FFA, which has a molecular structure close to FF, was discriminated. Then, the nanoMIP beads were tested with real samples of salmon and milk spiked with FF-HRP at 20–300 ng/mL. For the milk, with concentrations ranging at 60–80 ng/mL, a 95% recovery was obtained; whereas for the salmon (which has a more complex matrix), with concentrations ranging at 90–100 ng/mL, an 87% recovery was obtained, and for concentrations ranging at 150–300 ng/mL, a 77% recovery was obtained. These results were optimized by the extraction of FF to avoid the matrix effects. The nanoMIP beads also showed a good stability after six weeks of storage at RT and at 5 °C with 80.34% and 78.34% recovery for the milk samples and 71.48% and 74.3% recovery for the salmon samples, respectively [137]. Another example is toltrazuril (TZR), which is an anticoccidial (coccidian is an intestinal parasite) used for the treatment of coccidiosis in animal farming (poultry, cattle, sheep, and pig). TRZ residues are harmful to human health, and some regulations have fixed the limit of TRZ in food at 100–600 μg/kg of meat. Huang et al. used MIP immobilized on reduced graphene oxide (rGO) and titanium dioxide (TiO_2_)-modified platinum electrodes to detect toltrazuril in chicken muscles. MIP was prepared by mixing carboxymethyl-b-cyclodextrin (CM-b-CD) (monomer), TRZ (template), and tetraethylorthosilicate (TEOS) (crosslinker) following the optimal mole ratio of 2:1:30. NIP was fabricated the same way but without TRZ. The electrochemical sensor MIP/TiO_2_/rGO/Pt was fabricated by the successive coating of a Pt electrode with rGO/chitosan, TiO_2_/chitosan, and MIP/chitosan solutions. The template was removed by immersion in HCl (0.5 M) for 21 min. DPV was used to monitor the binding of TRZ in the imprinted cavities, and the optimal time for binding was determined as 16 min. The peak current decreased with an increase in TRZ concentration. The chicken muscles were treated with a high-speed blender, and then the extraction was performed with acetonitrile and sodium chloride, vortexed for 1 min, ultrasonicated for 20 min, centrifugated at 500 rpm for 10 min, and the supernatant was filtrated with a 0.22 μm filter. The response of the sensor was linear in the range of 0.43–42.54 ng/mL, and the LOD was 0.21 ng/mL (converted to ~0.63 μg/kg). The sensor was selective to TRZ in the presence of interferants (e.g., amprolium hydrochloride (ALDH), sulfaquinoxaline sodium (SQXS), and sulfamethazine (SMZ)), and the sensor’s response was reproducible with an RSD of 4.4% over six measurements and 5.2% with six different sensors. The sensor was also stable after 1 month at 4 °C with a drop of the current response of only 11% with 0.43 ng/mL of TRZ [138]. Tarannum et al. provided a review of the MIPs used for the detection of antibiotics in food [139].

### 2.2. Analytes in Human Cardiac Muscles

**Figure 7:**
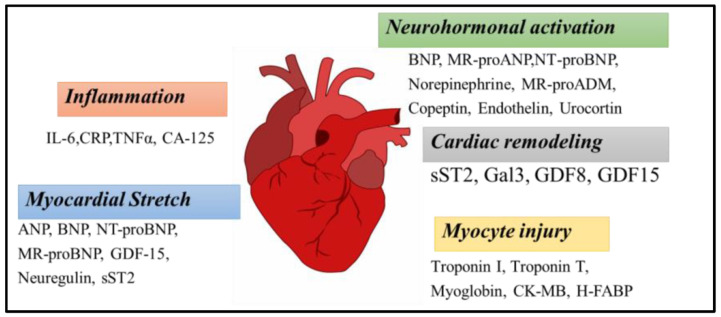
Schematic showing different cardiac muscle biomarkers. Reproduced from [140] with permission (open access).

Cardiovascular diseases encompass heart and circulatory diseases. Cardiac biomarkers (Figure 7) are extremely important from the point of view of acute myocardial infarction (AMI), because the sooner the diagnostic can be established, the better the patients will recover [140]. However, it is not easy to establish an AMI diagnosis, and several biomarkers and symptoms are needed for the precision of the diagnosis. An ideal biomarker should be easily accessible (noninvasively, if possible), sensitive, and specific to a normal or a pathogenic biological process, for which the measurement will be reproducible [141]. However, many biological molecules used as biomarkers are often not totally specific to a tissue, especially between skeletal muscles and cardiac muscles, which share several biomarkers [142,143].

- ***C-Reactive Protein***

C-reactive protein (CRP) is a ring-shaped pentameric protein of 120 kDa that is found in plasma and used as a biomarker of inflammation. The normal value of CRP in healthy individuals is less than 10 mg/mL, whereas during inflammation, this value can reach 350–400 mg/mL [144]. Cui et al. fabricated an MIP sensor (with an emphasis on reducing the nonspecific interactions) for the detection of human CRP in serum. The MIP was formed with the electropolymerization of a mixture of dopamine (monomer), PEG (monomer and antifouling), CRP (template), and graphdiyne (GDY) nanosheet (improved conductivity) at a ratio of 1:4:4:4 in Tris HCl at pH 8.5 and deposition on GCE. The template was removed by acetone 50 min. The binding of CRP in the imprinted cavities was monitored with EIS, with an optimized binding time of 5 min. The antifouling property of the MIP sensor was tested by immersion in fetal bovine serum (FBS ≤ 10%) for 30 min, and the change in the EIS evaluation (before and after immersion) was less than 5%, showing a good antifouling property due to the PEG. The sensor was tested with CRP at different concentrations in PBS (0.2 M, pH 7.4), and the sensor’s response was linear in the range of 10 fg/mL^−1^ mg/mL with an LOD of 4.1 fg/mL. The sensor was tested in the presence of interferants at increasing concentrations (e.g., immunoglobulin type G (IgG), alpha fetal protein (AFP), carcinoembryonic antigen (CEA), and transferrin (TRF)) and no obvious binding signal was generated by the interferants, whereas the sensor’s response in the presence of Na^+^, Cl^−^, K^+^, Ca^2+^, and Mg^2+^ at 10 ng/mL was negligible. The sensor also showed a long-term stability (over 25 days) and reusability, with only a slight change in its response after nine cycles of CRP binding/eluding. Then, the sensor was tested with CRP spiked in 10% human serum samples, and the RSD was mainly less than 5%. Furthermore, when tested with clinical blood samples, the sensor’s evaluation matched the clinical results within 7% [145]. This sensor had a wide range of linearity with a very low LOD. Although the binding time was only 5 min, the elution time was long (50 min). Furthermore, Balayan et al. fabricated an MIP sensor to detect CRP. An SPE-working electrode was modified with Au and Pt nanoparticles using electrodeposition. Then, the MIP was formed with the polymerization of a mixture of MMA (monomer), CRP (template), EGDMA (crosslinker), and AIBN in acetonitrile at 35 °C for 48 h. To remove the template, the MIP was washed in methanol/acetic acid for 24 h and with methanol only. The MIP was crushed into a fine powder and electrodeposited on the modified electrode with CV. The porosity of the MIP was evaluated using the Brunauer–Emmett–Teller (BET) method, and the MIP had a surface area of 9.358 m^2^/g, a pore volume of 0.032 cm^3^/g, and a pore diameter of 2.56 nm. The MIP/AuPt-NPs/SPE sensor was characterized using EIS and SWV, and measurement conditions were optimized at pH 6.5, and a temperature of 30 °C, with a response time of less than 5 min. The sensor’s response was linear in the range of 0.1–500 nM of CRP with an LOD of 0.1 nM. The sensor showed a high selectivity to CRP in the presence of interferants (e.g., glucose, uric acid, acetylcholine, cholesterol, ascorbic acid, serum amyloid A, TNFα, and procalcitonin). The reproducibility of the sensor was excellent with an RSD of 0.09% for the average current obtained with five different sensors tested [146]. In another study, Liu et al. fabricated a tungsten disulfide-doped peptide-imprinted polymer-coated electrode in an extended-gate field-effect transistor for the detection of CRP. Thus, MIP was electropolymerized on an ITO surface with a mixture of aniline (monomer), MSAN (monomer and dopant), WS_2_ (flake 90 nm, conductivity enhancer), peptide K (KESDTSYVSLKAPL) (template), and DI water. The template removal was obtained by washing with ethanol (5%) for 10 min at 130 rpm and then with DI water. The electrode was integrated into an extended-gate FET. The binding of CRP or peptide K in the imprinted cavities was monitored with CV. The sensor’s response was linear from 1 fg/mL to 1 ng/mL. The sensor was used to detect CRP in human blood serum in comparison with the results obtained with an ELISA kit. The results showed a 96% accuracy between the two techniques [147,148]. Finally, Chou et al. fabricated a thin film of MIP toward CRP by micro-contact printing. They used a cover glass coated with a functional monomer (o-(4-nitrophenylphosphoryl)choline) and template (CRP) to stamp a substrate coated with crosslinker (poly(ethylene glycol) 400 dimethacrylate, PEG400DMA). After UV (for 17 h), the cover glass was removed from the imprinted substrate, which was washed with SDS (2%)/NaOH (0.8%) at 80 °C for 30 min to remove the template. The MIP was immersed in a CRP solution (0.01 M) in PBS (pH 7.2) for 2 h, and the CRP rebinding was evaluated with ELISA after elution. The results showed a rebound amount of 3.78 ng/cm^2^ of CRP and 0.08 ng/cm^2^ of HAS used as a protein competitor [149].

- ***Heart-fatty acid binding protein (H-FABP)***

H-FABP is a cytoplasmic protein of 15 kDa, which is quickly released from cardiomyocytes during myocardial infarction. Its normal value in humans ranges from 0 to 2.8 ng/mL [150,151]. ST2 is a member of the Toll-like/interleukin 1 receptor family. There are two isoforms: the soluble sST2 and the transmembrane receptor ST2L. sST2 is involved in the regulation by the competition of the normal binding between IL-33 and ST2L. sST2 is considered as a biomarker of cardiac cell stress [152]. Crapnell et al. fabricated a nanoMIP sensor for the simultaneous detection of H-FABP and ST2. Glass beads were activated with NaOH (2 M) and functionalized with silane to obtain amine-conjugated beads, and templates (H-FABP and ST2) were immobilized on the modified glass beads by glutaraldehyde coupling [153]. NanoMIP was prepared using the radical polymerization of monomer and crosslinker (NIPAM, acrylic acid (AAc), N-tert-butylacrylamide (TBAm) and MBA) at RT for 2 h. The obtained product was eluted at a low temperature to remove the unreacted monomer and low-affinity nanoMIPs. Then, the high-affinity nanoMIPs was eluted at high temperature (60 °C) and harvested. The gold surface of an SPR chip was modified with mercaptoundecanoic acid, and the nanoMIPs were conjugated with EDC/NHS chemistry. They coupled thermal detection (HTM) with this multiplex nanoMIP sensor and detected H-FABP and ST2 in the physiological range with LODs of 4.18 and 8.79 ng/mL, respectively [154]. This study was the first to show a multiplex sensor allowing the simultaneous detection of two different compounds with thermal analysis.

- ***Heparin***

Heparin is a glycosaminoglycan (GAG) with an anticoagulant property by acting on anti-thrombin III [155]. Yoshimi et al. fabricated an MIP sensor for the detection of heparin. They grafted heparin-imprinted poly(methacryloxyethyltriammonium chloride-co-acrylamide-co-methylenebisacrylamide) on graphite particles. Template removal was obtained by washing with an aqueous solution of sodium chloride (1 M). Then, they mixed these modified graphite particles with silicon oil to fabricate an MIP-graphite paste (GP) and packed it into capillaries to fabricate MIP-GP electrodes. The MIP-GP electrode was used as a working electrode for analysis using CV at 0–8 unit/mL heparin in physiological saline or in bovine whole blood with 5 mM ferrocyanide. The current intensity increased with heparin, and the sensor allowed for the detection of heparin with great reproducibility [156]. In another study, Zhang et al. used pNIPAM to fabricate a thermoresponsive MIP sensor for the detection of heparin. They modified magnetic Fe_3_O_4_ nanoparticles with 3-methacryloxypropyltrimethoxysilane (MPS) at 50 °C overnight. MIP formed on the Fe_3_O_4_/MPS particles by the polymerization of a mixture of heparin disaccharide (template), NIPAM (thermoresponsive monomer), 2-aminoethylmethacrylate (AEM, positively charged monomer), AAm (monomer), MBA (crosslinker), ACVA (initiator), and water at 50 °C 24 h. The MIPs were collected with a magnet and washed with water. Template removal was obtained by washing with sodium chloride buffer (1 M) for 2 h. The MIPs were dried in a desiccator at 40 °C for 6 h. The rebinding experiments were performed at 25 °C, 35 °C, and 50 °C in the presence of heparin. Because of pNIPAM, the binding ability of the MIPs to heparin was regulated by the temperature. The results showed that, for the same amount of crosslinker (MBA 88%), the maximal binding capability was obtained at 25 °C and decreased with the increase in temperature due to the shrinking of pNIPAM at temperatures over the lower critical solution temperature (LCST 32 °C). Heparin was eluted from MIPs in a tube, the supernatant was collected, and the heparin concentration was evaluated by colorimetry with the carbazole reaction on uronic acid units of the heparin. The imprinting factor was 3.2 [157]. Furthermore, readers interested by this class of responsive MIP sensors can refer to the review in [48].

- ***Metoprolol***

Metoprolol (1-(4-(2-methoxyethyl)phenoxy)-3-(propan-2-ylamino)-2-propanol) is a β-blocker used in cardiac disease and high blood pressure to reduce the heart beat rate. Gungor et al. fabricated an MIP sensor to detect metoprolol with a p(AN-co-PTSA)-modified GCE obtained by electropolymerization (5 cycles, 100 mV/s) of a mixture of aniline, and p-toluene sulfonic acid (PTSA) in PBS at optimized pH 1.6. The template was removed by immersion in HCl (0.1 M) for 24 h. The binding of metoprolol to the imprinted cavities was monitored with SWV. The response of the sensor was linear in the range of 40–1500 μM with an LOD of 37.9 μM. The reproducibility of the sensor over ten measurements with the same sensor was high, with an RSD of 2.53% [158]. In another study, Khan et al. fabricated an MIP-TiO_2_-based carbon paste electrode for the detection of metoprolol. The MIP was prepared with the bulk polymerization of a mixture of MAA (monomer), metoprolol (template), EGDMA (crosslinker), and AIBN (initiator) in chloroform in a water bath 60 °C for 18 h. The template removal was obtained using Soxhlet extraction (methanol: acetic acid at 70:30) overnight. An electrode of TiO_2_/MIP/CPE was prepared by mixing MIP nanoparticles, graphite powder, TiO_2_, and n-eicosane as a binder. The binding of metoprolol to the imprinted cavities was monitored by chronoamperometry and CV. The sensor’s response was linear in the range 10–120 μM with an LOD of 5 μM. No current increase was observed when the sensor was tested in the presence of interferants (e.g., tryptophan, glucose, urea, ascorbic acid, and uric acid). A metoprolol tartrate tablet was dissolved in phosphate buffer at pH 7, different concentrations were tested on the sensor, and the recovery rate range was between 96 and 102% [159]. Other cardiovascular drugs, such as timolol, minoxidil, and bisoprolol fumarate, have been used as templates for the fabrication of MIP sensors and described in an interesting review on MIP-modified carbon paste electrode (CPE), as reviewed by Asfharara et al. [160].

- ***Myoglobin***

Myoglobin is released into the blood stream within the first few hours when the heart or skeletal muscles are injured; therefore, it is a potential biomarker for detecting acute myocardial infarction. Ribeiro et al. used SPE and MIP for the detection of myoglobin. They electropolymerized a solution of phenol and myoglobin in PBS (0.1 M) at pH 7 on Au SPEs. The polyphenol-deposited film had a thickness of 4.4 nm. The template was removed by immersion in SDS in PBS/methanol (10:1) solution overnight. The binding of the template to the imprinted cavities with incubation for 10 min was monitored by SWV in the presence of ferro/ferricyanide and the redox peak current decreased with the increase in Myo 0.001 ng/mL–100 μg/mL (linear range). The LOD was 2.1 pg/mL when evaluated in PBS and 14 pg/mL when evaluated in artificial serum, which is below the threshold needed for the detection of a myocardial infarction (myoglobin: 70–200 ng/mL). The SPE MIP sensor was also selective to myoglobin when compared to the SPE NIP with eight-fold more affinity [161]. Similarly, Moreira et al. immobilized myoglobin by adsorption on Au SPE in acetate buffer at pH 5 for 15 min. Then, they electropolymerized a solution of o-aminophenol (monomer) on myoglobin (template) in acetate buffer at pH 5. Template removal was obtained by immersion in proteinase K solution in PBS at pH 7.4 overnight. Using SWV, the Au SPE/Myo/PAP sensor showed a linear response down to 2.22 μg/mL of myoglobin with an LOD of 0.827 μg/mL [162]. In another study by the same group, the carboxylic groups of oxidized graphite were activated using N-ethyl, N′-(3-dimethylaminopropyl) carbodiimide hydrochloride (EDAC)/NHS and reacted with myoglobin (0.1 mM) in PBS at pH 7, for 2.5 h at 4 °C. After washing, the MIP formed on the myoglobin-graphite particles with the polymerization of a mixture of 4-styrenesulfonic acid sodium salt (SSA), 2-aminoethyl methacrylate hydrochloride (AEHM, monomer), benzoyl peroxide (BPO, initiator), and EDGMA (crosslinker) at 38 °C for 3 h. The template was removed by immersion in a solution of acetic acid (10%)/SDS (10%) for 2 h. The MIP/graphite particles were included in a myoglobine-selective membrane fabricated by mixing them in a mixture of PVC and plasticizer o-nitrophenyloctylether (o-NPOE). Then, this membrane was casted on the working electrode of an SPE. The sensor was evaluated with SWV in the presence of ferricyanide at pH 5. The peak current decreased with the increase in myoglobin. The sensor’s response was linear in the range of 0.2–1.8 μg/mL of myoglobin with an LOD of 0.79 μg/mL. The sensor was tested with the interferants (e.g., BSA, creatinine, hemoglobin, and NaCl), and a good selectivity to myoglobin was observed. Then, the sensor was tested with spiked urine samples with myoglobin, and the RSD was 1.8% [163]. In another study, Shumyantseva et al. electropolymerized a solution of o-PD (monomer) and myoglobin (template) with a ratio of 10:1 in potassium phosphate buffer at pH 7.4 on SPE with a graphite working electrode. The template was removed by immersion in ethanol/water (2:1) with NaOH (0.25 M) for 15 min at 50 °C [164]. The myoglobin binding to the imprinted cavities was monitored with SWV. The sensor had a linear response in the range of 1 nM–1 mM with an LOD of 0.5 nM (9 ng/mL). The sensor showed selectivity to myoglobin with an IF of 2–4. Moreover, when tested in the presence of interferants (e.g., cytochrome c, lysozyme, and human serum albumin), the response to myoglobin was significantly higher compared to the response obtained with interferants. The sensor was tested in human serum samples spiked with myoglobin and in plasma samples from patients with AMI and from healthy donors, and the three groups were clearly discriminated. Especially, myoglobin in the plasma from patients with AMI was evaluated in the range of 15–25 nM, while myoglobin from the plasma of healthy donors was evaluated in the range of 2–5 nM [165]. In another study, Alhmeizia et al. fabricated a potentiometric MIP sensor for the detection of myoglobin. To this end, myoglobin was attached to carboxylated multiwalled carbon nanotubes (MWNTs-COOH) with EDAC/NHS chemistry. After blocking the unreacted ester groups by immersion in TRIS buffer (1 M) for 30 min and rinsing with water, a polymerization mixture of AAm (monomer) and MBA (crosslinker) in a piperazine-N,N-bis(2-ethanesulfonic acid) (PIPES) solution at pH 7 was added to the myoglobin-MWNTs. After 45 min in the polymerization mixture, APS (initiator) was added, and the polymerization started for 1 h at RT. Then, the MIP was washed with water. The template was removed with the incubation of the MIP with oxalic acid (1 M) for 12 h. Electrodes were fabricated on a hydrophobic paper substrate coated with CF^10^ (fluorinated alkyl silane, CF_3_(CF_2_)_7_CH_2_CH_2_SiCl_3_) using stencil masks and carbon paste. A reference electrode Ag/AgCl was printed on the CF^10^-coated paper. MIP particles were dissolved in tetrahydrofurane (THF) with poly(vinyl chloride) (PVC) and o-nitro-phenyloctyl ether (o-NPOE), and 20 mL of the mixture was drop-casted on the carbon paste electrode and let to dry to form the working electrode. Potentiometric measurements were performed with a potentiostat in HEPES buffer (10 mM) at pH 4. Different concentrations of myoglobin (from 10 nM to 1 mM) were tested, and the MIP sensor’s response was linear with the logarithm of the myoglobin concentration in the range of 100 nM–0.1 mM and with an LOD of 100 nM. The MIP sensor was tested with numerous interferants (e.g., creatinine, sucrose, fructose, galactose, sodium glutamate, thiamine, alanine, ammonium, uric acid, albumin, glutamine, guanine, troponin T, and glucose) and their influence was minimal. The MIP sensor was also tested with spiked samples of artificial serum and of real blood. For the five spiked artificial serum samples, the recovery ranged from 93% to 103% with an average RSD of 4.5%, and for the four spiked real blood samples, the results were comparable with those obtained with a conventional method [166].

- ***Troponin***

Troponin is a protein involved in the regulation of skeletal and cardiac cell contraction via calcium binding. The cardiac troponin T (cTnT) level in the blood stream of healthy patients is less than 10 pg/mL and increases to over 10 pg/mL in AMI [167]. Phonklam et al. fabricated an MIP sensor for the detection of cTnT. First, SPCEs were modified with 20 μg functionalized MWCNTs in dimethyl formamide (DMF) and dried at 35 °C for 30 min. Then, methylene blue was electrodeposited on the surface of the modified SPCE to obtain polymethylene blue (PMB)/fMWCNTs/SPCE. An MIP coating on the surface of the modified electrode was formed by the electrodeposition of aniline in the presence of HCl, followed by cTnT (template) drop-casted onto the surface and left overnight at 4 °C and after washing by a second electrodeposition of aniline in the presence of LiClO_4_ to obtain cTnT-PANI/PMB)/fMWCNTs/SPCE. Template removal was obtained by immersion in acetic acid (0.5 M) for 4 h. The binding of cTnT in the imprinted cavities was monitored by DPV with a time of 30 min used for target binding. The sensor showed a linear response in the range of 0.1–8 pg/mL with an LOD of 0.04 pg/mL. The sensor was tested with interfering compounds (e.g., cardiac troponin I (cTnI), glucose, ascorbic acid, creatinine, uric acid, and human serum albumin), and no interfering effect was observed. The sensitivity of six different MIP sensors was evaluated, and the RSD was 1.4%, whereas the long-term stability of an MIP sensor was good, with a sensitivity > 90% after 6 weeks at RT and 83.2% after 8 weeks of storage. Human blood plasma samples were diluted 100 times, and the cTnT was evaluated with the MIP sensor and with an electrochemilluminescence immunoassay. The results showed excellent agreement between the techniques [57]. Furthermore, Palladino et al. fabricated an MIP sensor using epitope imprinting for the detection of troponin (TnT). A polydopamine film formed on a gold-coated-SPR surface using dopamine (monomer), TnT (template) (TnT protein, or synthetic peptides residues 1–10 (P1: MSDIEEVVEE), 50–60 (P2: EEAKEAEDGPM), 136–150 (P3: EQQRIRNEREKERQN), and 279–288 (P4: GKAKVTGRWK)), and TRIS HCl pH 8.5 at 25 °C for 5 h. The removal of the template was obtained by washing with 5% acetic acid. For the MIP sensor made with the whole TnT template, the rebinding experiment was performed with a binding time of 2 min. The sensor’s response was linear in the range of 0–289 nM with an LOD of 15.4 nM. The sensor was selective to TnT with a negligible interaction of the interferant HAS with MIP and NIP. For the MIP sensor made with the four synthetic peptides used as a template (MIP-1234), the linear response was in the range of 0–289 nM of TnT with a higher sensitivity and lower LOD (14.8 nM). Interestingly, when MIP-1234 was tested with each synthetic peptide separately (instead of TnT), the rebinding experiment showed that only the synthetic peptide P4 corresponding to TnT C-terminus was recognized by the sensor. This result was confirmed by making the MIP sensors (MIP-1, MIP-2, MIP-3, and MIP-4) with only one synthetic peptide used as a template. The rebinding experiment with TnT showed that only MIP-4 was efficient at detecting TnT with an LOD of 41 nM [45].

The troponin cTnI isoform in the blood stream is usually less than 0.04 ng/mL and rises to 1.4 ng/mL within 3–12 h during AMI [168]. Choudhary and Altintas developed a portable nanoMIP-SPRsensor for the detection of cardiac troponin I (cTnI). They used the solid-phase synthesis method. First, they functionalized glass beads by boiling them in NaOH (2 M) for 15 min, salinizing by incubation in 2% APTES in toluene for 24 h at RT, and then immersion in 7% glutaraldehyde in PBS for 2 h at RT to obtain aldehyde-functionalized glass beads. Then, they conjugated the epitope template (cTnI-derived small-peptide ISASRKLQLK) by incubation with the modified glass beads in PBS overnight at 4 °C. After washing with water, the beads were incubated in NaBH_4_ solution in PBS at RT for 30 min to reduce the Schiff bases and then immersed in ethanolamine solution in PBS for 15 min to block the peptide-free surface. Second, the nanoMIPs were formed on the template-conjugated beads with the polymerization of a mixture composed of N-isopropyl acrylamide (monomer), MBA (crosslinker), and N-(3-aminopropyl)methacrylamide hydrochloride, and then N-tert-butyl acrylamide, methacryloxyethyl and thiocarbamoyl rhodamine B were added to APS (initiator) and TEMED (activator) for 2 h at RT. The beads were washed with cold water (15 °C) to remove the unreacted monomer and products and low-affinity nanoMIPs. Then, the beads were washed with hot water (65 °C) to elude and harvest the high-affinity nanoMIPs free of the template. The nanoMIPs obtained had a size of 155.7 nm with a very-low polydispersity index of 0.1543, as evaluated with dynamic light scattering (DLS). Third, they immobilized the nanoMIPs on the gold-coated surface of a commercial SPR sensor, with a thin film of 11-mercaptoundecanoic acid by the activation of the carboxyl groups with EDC/NHS for amine coupling. The binding of cTnI to the imprinted cavities was monitored by SPR measurements. The sensor’s response was linear in the range of 0.78–50 ng/mL of cTnI with an LOD of 0.52 ng/mL. The nanoMIP sensor showed a high selectivity to cTnI in the presence of interferants (e.g., glucose, BSA, transferrin, and p53 protein) [169]. Moreover, McClements et al. fabricated nanoMIPs (size 71 nm) for the detection of cTnI and studied the effects of the method used (dip coating, drop casting, and covalent functionalization) to immobilize nanoMIPs onto SPE on the sensing capabilities. Thus, nanoMIPs were physiosorbed on a type K thermocouple by dip coating for 1 min in an aqueous nanoMIPs solution and by being let to dry for 2 h at RT. The nanoMIPs were also physiosorbed on an SPE by the drop casting of 8 μL of a nanoMIP solution and then being let to dry for 16 h. The nanoMIPs were also conjugated with EDC/NHS chemistry on an SPE-modified surface. The nanoMIP-modified thermocouples and SPE were placed in 3D printed flow cells with PBS for heat transfer measurements in the presence of cTnI (0–50 ng/mL) in PBS. The results showed that, with the dip-coated thermocouple, a linear response was observed in the range of 0–10 pg/mL of cTnI with an LOD of 0.55 pg/mL and a high specificity for cTnI. With the drop-casted SPE with cTnI (0.005–1 pg/mL) in PBS, the LOD obtained was 0.01 pg/mL but varied to 0.03 pg/mL over triplicate measurements. With the nanoMIP-functionalized SPE and cTnI (0.1–1 pg/mL), the LOD obtained was 0.46 pg/mL [170]. The deposition of the MIP on the transducer during the sensor’s fabrication is of great importance, and Caldara et al. wrote an interesting review on this topic [69]. Similarly, Zuo et al. fabricated an MIP electrochemical sensor for the detection of cTnI by electrodeposition of a solution of o-amino-phenol, cTnI (template), and NaClO_4_ at pH 7 on a bare carbon electrode. After the removal of the template with PBS, the detection of cTnI in PBS containing 1 mM K_3_[Fe(CN)6] was monitored with DPV. The sensor’s response showed linearity in the range of 0.05–5 nM with an LOD of 0.027 nM and a detection time within 5 min. Furthermore, the sensor showed a high selectivity against cTnI when compared to the structural analog bovine serum albumin (BSA), and when tested with human serum, the sensor was able to detect cTnI in the range of 1–100 nM [171]. In another study, Baldoneschi et al. fabricated an MIP SPR sensor for the detection of cTnI by epitope imprinting of the C-terminus peptide (residues 197–210, ALSGMEGRKKKFES) and N-terminus peptide (residues 28–40, AYATEPHAKKKSK) of cTnI. A bare gold SPR chip was coated with norepinephrine film by drop casting in 20 mM Tris-HCl at pH 8.5 in the presence of C-terminal and N-terminal peptides (template) (MIP) or not (NIP) for 5 h at 25 °C. After template removal with acetic acid (5% in water), cTnI (in PBS at pH 7.4) binding to the imprinted cavities was evaluated with an SPR-based refractometer. The sensor’s response was linear in the range of 0–20 nM cTnI with an LOD of 0.46 nM. The MIP sensor was tested with interferants (e.g., TnT and HSA) and three types of mouse monoclonal antibodies against TnI (TPC110, 19C7, and MF4) and showed a high selectivity to cTnI. Then, cTnI from plasma samples was extracted with streptavidin-coated magnetic microbeads functionalized with biotinylated anti-cTnI antibodies and injected on the sensor. The LOD increased to 8.9 nM [172]. Table 1 summarizes the MIP sensors fabricated for the detection of the different analytes relevant to the skeletal and cardiac muscles that we described.

### 2.3. Commercial Potential of MIP Sensors for the Detection of Skeletal and Cardiac Muscle Analytes

An important demand exists in the biomedical field for biosensors in general and especially for MIP sensors. The market for biosensors in the US was predicted 10 years ago to be USD 17 billion in 2018 [173]; currently, it is valued at USD 28.9 billion in 2023 [174] and it is expected to grow to USD 49.78 billion in 2030. Several companies and start-ups manufacturing MIP sensors exist in different countries, such as Sigma-Aldrich (USA), Affinisep (France), MIP Diagnostic Ltd. (UK), Sixth Wave Innovation Inc (Canada), Semorex (Israel), and Aspira Biosystems (USA). MIP sensors have many advantages that are in favor to their commercialization. They are low cost, resistant to harsh conditions (pH, temperature, and pressure), have high selectivity, can have ultra-low LOD, and have a large spectrum of materials and methods for their fabrication, which provides choices for their translation into mass production. However, some studies pointed out that, despite numerous patents filed worldwide on MIPs, the technology still remains in laboratories with few exceptions, such as solid phase extraction materials based on MIP by Sigma-Aldrich and other companies [175,176]. Although the market is large with different fields of applications, such as the environment, the control of food, and the biomedical field, currently, the commercialization of MIP sensors is only focused on certain market niches [175]. We inform readers of a recent book chapter on the topic of MIP and commercialization by Singh [177]. This is also visible in the research for the detection of skeletal and cardiac muscle analytes, where many MIP sensors are developed for acute myocardial infarction, for example, while for other pathologies, this development is not yet conducted. In addition, there are differences depending on who is targeted in the market, whether it is general public or professionals (e.g., clinics, hospitals, and companies). For the detection of skeletal and cardiac muscle analytes with MIP sensors, the first target is professionals. Moreover, there are some technical aspects that seem to have to be overcome before the real commercialization of MIP sensors. The main challenge is to fabricate MIPs in large batches that are homogeneous in size, shape, and affinities [175,176]. Furthermore, MIP sensors will be used with real complex matrix samples and should be able to be sensitive despite the interferences [175]. Moreover, the development of a friendly read out via smartphone or others is needed. In addition, the development of MIP sensors for mass production faces the normal difficulties encountered for the development of news products, which are the needs for the reengineering of the fabrication process to be conformed to the industrial good manufacturing process, and the need to be conformed to some regulations. However, we can consider that MIP sensors are destinated to be used out of the human body, they do not interfere with the human health, and therefore the regulation should be less severe [178].

## 3. Conclusions

The development of MIP-based biosensors is a very dynamic field that makes it difficult to cover exhaustively in a review. Instead, this review provided an overview of MIP sensors used to detect analytes related to skeletal and cardiac muscles. The goal was to highlight the different types of MIP sensors and to gather as many differently targeted biomarkers related to skeletal and cardiac muscles as possible. When possible, we favored the most recent papers without delving too deeply on a given analyte.

As can be seen from this review, a great variety of MIP sensors for skeletal and cardiac muscle analytes have been fabricated, and different imprinting techniques (e.g., bulk imprinting, solid-phase imprinting, nanoimprinting, and epitope imprinting) and different formats (e.g., impedimetric, potentiometric, and colorimetric) have been used for their fabrication. The main characteristic of these MIP sensors is their specificity, which is usually high because of the MIP used as the recognition element. Furthermore, most of these MIP sensors also showed good reproducibility, repeatability, could be reused, and had a good shelf life without specific environmental conditions. Interestingly, some MIP sensors reached a very-low LOD (femtomolar order). A few examples also showed multiplex sensing. In terms of difficulties, a few MIP sensors sometimes showed the formation of nonuniform binding sites, which was observed through the linear response of these sensors that was composed of two segments with different slopes. In addition, for others MIP sensors, the time used to remove the template and the rebinding times were long. For medical applications, we expect MIP sensors to be fast in their analysis, easy to use, non-bulky, and, if possible, portable. Interestingly, most of these MIP sensors were tested with real samples (blood, urine, and sweat) and were efficient in their detection and response. However, the complexity of such matrices is usually a common challenge for sensor detection, and this problem is usually overcome by diluting the samples. In an optimized process setting, it would be good if the MIP sensors could be used directly on samples without these additional dilution steps.

As we can see, the development of MIP sensors for the detection of skeletal- and cardiac-muscle-related analytes is already substantial, and many different biomarkers have been used. However, this field needs more development. MIP sensors for the detection of skeletal- and cardiac-muscle-related analytes is important not only for diagnosis, but also for drug discovery, self-monitoring, and for the evaluation of the functionality of engineered skeletal and cardiac tissues. Currently, we can observe from the literature that the development of such MIP sensors is more substantial for applications in well-known and important pathologies, such as acute myocardial infarction (AMI) and cancer, but for other pathologies, the literature is more diffuse with less fabricated MIP sensors.

To enhance the development of MIP sensors for skeletal and cardiac tissues, biological and engineering knowledge must be combined. The first thing to understand is the different biomarkers that can be used for imprinting in the fabrication of MIP sensors and for what myopathy or biological conditions these biomarkers are characteristic. The best biomarkers are specific to one tissue and sensitive to the biological state. This knowledge can be obtained by new research development in the field of biomarkers, by enhancing communication between clinical staff and biomedical engineers, and by papers, such as the present one, which lists different biomarkers. By presenting the different sensors through their targeted analytes, we hope this review can contribute to enhancing the development of MIP sensors in the field of skeletal and cardiac muscles.

## Figures and Tables

**Table 1:** MIP sensors used to detect skeletal- and cardiac-muscle-related analytes.

Analytes	Materials	Analytical Technique	Linear Range	Limit of Detection	Ref.
**Analytes in human skeletal muscle**					
baclofen	SiNPs/MBA/PEG400/1,3-propane sultone	Spectropho-tometry	0.1–1 mg/mL	0.125 μM	[50]
bisphenol A	MAA/ZnFe_2_O_4_/paper	colorimetry	10 nM–1 μM	6.18 nM	[82]
bromisoval	NiO/NiFe_2_O_4_@Zn_0.76_Co_0.24_S/GCE	DPV	6 nM–3.7 μM	0.28 nM	[85]
creatinine	AMPS/MBA/hexadecanethiol-coated gold	EIS	0–600 μM	10 μM	[92]
creatinine	MAA/EGDMA/Ag2S-QDs	spectrophometry	0.15–0.5 mg/mL	0.6 ng/mL	[70]
creatinine and 8-OHdG	Graphite-PDMS/CuO-MIP NPs/& PtNPs-rGO-MIP	amperometry	0.5–150 μM creatinine 5 nM–50 μM (8-OHdG)	77 nM (creatinine) 0.8 nM (8-OHdG)	[89]
creatinine-Cu (II)	CuBr_2_/MAA/EGDMA/Carbon paste electrode (MIP-CPE)	CV	0.1–10 μM	59 nM	[90]
glucose	MIP-PMMA on stub resonator	frequency GHz	0.5–4 mg/mL	24 pg/mL (LLD)	[93]
glucose	AuNPs-MIP	Amperometry, CV, SWV	1.25–320 nM	1.25 nM	[94]
glucose	Elelectrospun nanofiber (nylon 6,6 PPY particles)-MIP	HTM method	0–0.8 mM	0.12 nM	[95]
glucose	Dummy MIP particles/PVC/Al plate	HTM method	19–330 μM in PBS, 44.4–330 μM in urine	19.4 μM (PBS), 44.4 μM (urine)	[72]
glutathione	MAA/EDMA/thin film/graphite	potentiometry	10–200 μM	N/A	[99]
growth hormone	MIP/Fe_3_O_4_ NPs/GCE	SWV	0.1–100 ng/mL	0.06 ng/mL	[100]
insulin	MIP cryogel/f-MWCNTs/AuE	SWV	50 fM–1.4 pM	33 fM	[102]
insulin	MIP/SPCE	SWV	20–70 pM	1.9 pM	[103]
insulin	MIP (PHEMAH)/QCM	QCM	1.38 pM–1.72 nM	0.27 pM	[104]
IL-1b	MIP/SPCE	EIS	0.1 pg/mL–1.05 ng/mL	0.23 pg/mL	[106]
IL-6	APBA/Pt/Polycarbonate microneedles	EIS	>1 pg/mL	1 pg/mL (LLD)	[109]
IL-6	MIP(pDa)/PNT/SPE	EIS	1–200 pg/mL	0.25 pg/mL	[110]
IL-6	MIP/C-SPE	EIS	0.02 pg/mL–20 ng/mL	0.1 pg/mL	[111]
lactate	MIP/Ag-Au NPs/SPCE	amperometry	1–220 μM	3 nM	[113]
lactate	MIP/rGO-AgNPs/AuE	CV	10–250 mM	0.726 mM	[114]
lactate	MAA/EGDMA	spetrophotometry	0.1–1.7 mM	162 μM	[115]
lactate	MIP/AuNPs/rGO/GCE	DPV	0.1–15 nM	0.09 nM	[116]
lactate	PANI/PBA/pNIPAM/(CNC/CNT)	CV	1–25 mM	0.1 mM	[117]
lactate	PANI-co-PBA/AuNP@PANI/CNT/CNC/AgNP@PDMS/CNT/CNC	CV	25–150 mM	0.07 mM	[118]
lactate	MIP-NPs/MWCNTs/PVC membrane	potentiometry	1 mM–0.1 M	0.733 mM	[119]
lactate	Poly(3-APBA)/SPE	CV	3–100 mM	1.5 mM	[120]
myoglobin	MIP/MPA/QDs(CdTe)	fluorescence spectroscopy	50.6 fM–95 pM	7.6 fM	[121]
myoglobin	MIP/DSP/SPE	EIS	1–20 ng/mL	0.83 ng/mL	[122]
myoglobin	Poly(HEMA-MATrp)/(gold)SPR	SPR	0.1–1 mg/mL	26.3 ng/mL	[125]
myostatin	(norepinephrine)/(gold)SPR	SPR	0.63–10 mg/mL	70 ng/mL	[126]
ractopamine	PDA/Apt/RAC/AgNPs/CGE	EIS	1 fM–1902 nM	330 aM	[127]
testosterone	MIP(p(HEMA-MAA))/(gold)SPR	SPR	0.5–20 ng/mL	49 pg/mL	[129]
testosterone	MIP/ITO	CV	0.1–100 pg/mL	2 pg/mL	[130]
testosterone	MIP(PANI-co-MSAN)/SPE	CV	1–1000 ag/mL	10 ag/mL	[131]
nandrolone	MIP/Au/GCE	amperometry	5–95 mM	3 nM	[133]
metandienone	MIP/GO/GCE	amperometry	0–2900 ng/mL	70 pM	[134]
stanozolone	MIP/CNTs/GCE	amperometry	0–120 mM	9 pg/mL	[135]
**Analytes in animal skeletal muscles**					
enrofloxacin	MIP/AgNPs/copper rods	Raman spectroscopy	1–100 ng/mL	0.025 ng/mL	[136]
florfenicol	MIP/glass beads	spetrophotometry	10–300 ng/mL	<10 ng/mL	[137]
toltrazuril	MIP/TiO_2_/rGO/Pt	DPV	0.43–42 ng/mL	0.21 ng/mL	[138]
**Analytes in human cardiac muscles**					
CRP	MIP (graphydine)/GCE	EIS	10 fg/mL–1 mg/mL	4.1 fg/mL	[145]
CRP	MIP/AuPt-NPs/SPCE	EIS and SWV	0.1–500 nM	0.1 nM	[146]
CRP	MIP((PANI-co-MSAN)WS_2_)/	CV	1 fg/mL–1 ng/mL	N/A	[147]
CRP	MIP	ELISA (after elution)	3.78 ng/cm^2^	N/A	[149]
H-FABP	MIP/(gold)SPR	HTM method	N/A	4.18 ng/mL	[154]
Heparin	MIP/graphite paste	CV	0–8 unit/mL	N/A	[156]
Heparin	MIP (with NIPAM)/Fe_3_O_4_/MPS	colorimetry	30–180 ng/mL	N/A	[157]
Metoprolol	p(AN-co-PTSA)/GCE	SWV	40–1500 mM	37.9 mM	[158]
Metoprolol	TiO_2_/MIP/CPE	CV	10–120 mM	5 mM	[159]
Myoglobin	MIP(polyphenol)/Au/SPE	SWV	1 pg/mL–100 mg/mL	2.1 pg/mL	[161]
Myoglobin	Myo/PAP/Au-SPE	SWV	>2.22 μg/mL	0.827 mg/mL	[162]
Myoglobin	PVC-o-NPOE-MIP/GO/SPE	SWV	0.2–1.8 mg/mL	0.79 mg/mL	[163]
Myoglobin	o-PD/SPE	SWV	1 nM–1 mM	0.5 nM (9 ng/mL)	[165]
Myoglobin	MIP/MWNTs/carbon paste electrode	potentiometry	0.1 mM–0.1 mM	0.1 mM	[166]
Troponin T	PANI/PMB/MWCNTs/SPCE	DPV	0.1–8 pg/mL	40 fg/mL	[57]
Troponin T	MIP (PDA)/(gold)SPR	SPR	0–289 nM	15.4 nM	[45]
Troponin I	MIP/MUDA/(gold)SPR	SPR	0.78–50 ng/mL	0.52 ng/mL	[169]
Troponin I	nanoMIP/thermocouple	HTM	0–10 pg/mL	0.55 pg/mL	[170]
	nanoMIP/SPE (by drop casting)	HTM	0.005–1 pg/mL	0.01 pg/mL	
	nanoMIP/SPE (electrografting)	HTM	0.1–1 pg/mL	0.46 pg/mL	
Troponin I	MIP(o-aminophenol)/GCE	DPV	0.05–5 nM	0.027 nM	[171]
Troponin I	MIP(norepinephrine)/(gold)SPR	SPR	0–20 nM	0.46 nM	[172]

## Data Availability

Not applicable.
